# Crosslinking Induced Reassembly of Multiblock Polymers: Addressing the Dilemma of Stability and Responsivity

**DOI:** 10.1002/advs.201902701

**Published:** 2020-03-06

**Authors:** Rui Yang, Yi Zheng, Xiaoyu Shuai, Fan Fan, Xueling He, Mingming Ding, Jianshu Li, Hong Tan, Qiang Fu

**Affiliations:** ^1^ College of Polymer Science and Engineering State Key Laboratory of Polymer Materials Engineering Sichuan University Chengdu 610065 China; ^2^ Laboratory Animal Center of Sichuan University Chengdu 610041 China

**Keywords:** click chemistry, crosslinking induced reassembly, drug delivery, fluorescence resonance energy transfer, multiblock polyurethane

## Abstract

Physical or chemical crosslinking of polymeric micelles has emerged as a straightforward approach to overcome the intrinsic instability of assemblies. However, the crosslinking process may compromise the responsivity of nanosystems and result in inefficient release of payloads. To address this dilemma, a crosslinking induced reassembly (CIRA) strategy is reported here to simultaneously increase the kinetic and thermodynamic stability and redox‐responsivity of polymeric micelles. It is found that the click crosslinking of a model multiblock polyurethane at the micellar interface induces microphase separation between the soft and hard segments. The aggregation of hard domains gathers liable disulfide linkages around the interlayer of micelles, which could facilitate the attack of reducing agents and act as an intelligent on‐off switch for high stability and triggered release. As a result, the CIRA approach enables an enhanced tumor targeting, improved biodistribution and excellent therapeutic efficacy in vivo. This work provides a facile and versatile platform for controlled delivery applications.

## Graphical abstract

Crosslinking induced reassembly (CIRA) of multiblock polyurethanes induces a microphase separation between the soft and hard segments and gathers liable disulfide linkages around the interlayer of micelles, thus leading to a simultaneous improvement of stability and responsivity of assemblies. The CIRA strategy enables an intelligent on‐off switch for enhanced tumor targeting, improved biodistribution and excellent therapeutic efficacy in vivo.

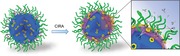

Self‐assembled polymeric micelles have been receiving much attention for potential use as versatile drug delivery systems.^[^ ^1^, ^2^, ^3^ ^]^ They commonly consist of hydrophobic cores for protecting and solubilizing therapeutics and hydrophilic shells for improved colloidal stability and stealthy character.^[^ ^4^ ^]^ To date, a good variety of polymer micelle‐based nanomedicines (Genexol‐PM,^[^ ^5^ ^]^ NC‐6004,^[^ ^6^ ^]^ NK105,^[^ ^7^ ^]^ NK012,^[^ ^6^ ^]^ and NK911,^[^ ^8^ ^]^ etc.) have been approved by Food and Drug Administration (FDA) or entered the clinical trials. Disappointingly, their therapeutic effect does not meet the expectations, as evidenced by the limited clinical outcome.^[^ ^9^ ^]^ A principal challenge is that most polymeric assemblies cannot withstand the massive dilution and competing interactions with blood components due to their dynamic nature and intrinsic instability, leading to premature burst release of drugs and nonspecific biodistribution in vivo.^[^ ^10^ ^]^

To overcome this limitation, physical or chemical crosslinking of the core,^[^ ^11^, ^12^ ^]^ shell^[^ ^13^, ^14^ ^]^ or interlayer^[^ ^15^ ^]^ of polymeric micelles has emerged as a straightforward approach to stabilize the assembled structures, assuring a prolonged circulation time without disassembly‐induced drug leakage.^[^ ^16^ ^]^ However, overly stable nanocarriers are also problematic, since slow and inadequate release of therapeutics may result in an insufficient intracellular drug availability for killing cancer cells and potential induction of multidrug resistance (MDR).^[^ ^17^, ^18^ ^]^ Under these circumstances, researchers have developed various cleavable linkers^[^ ^19^, ^20^ ^]^ or physical interactions^[^ ^21^ ^]^ to construct reversibly stabilized micelles. Nonetheless, these systems still cannot release drugs efficiently because a decrosslinking process should be involved before release of payloads from their cores. This two‐stage process limits their prospects and, it seems clear that increasing the stability of polymeric micelles while ensuring the efficiency of drug release are two conflicting purposes.

To address the dilemma, here we propose a crosslinking induced reassembly (CIRA) strategy to simultaneously increase the stability and specific drug release rate of polymeric micelles, taking multiblock polyurethane (MPU) as a model. MPU has been established as a leading polymer for implants, tissue engineering, and drug delivery applications.^[^ ^22^ ^]^ With excellent molecular tunability and unique phase behavior, MPU provides an outstanding platform allowing for facile control of self‐assembly properties. We and others have recently reported a series of MPUs with controllable micellization, multimodal targeting and smart response properties for on‐demand delivery of therapeutics and imaging agents.^[^ ^23^, ^24^, ^25^ ^]^ However, to the best of our knowledge, MPU formulations with simultaneous improvement of stability and responsivity in vivo have not yet been developed.

To validate our basic concept of CIRA, we first synthesized a model clickable multiblock multifunctional polyurethane. The polymer was constructed from biodegradable poly(ε‐caprolactone) (PCL), cleavable polyethylene glycol bearing a pH‐responsive benzoic‐imine linkage (BPEG), L‐lysine ethyl ester diisocyanate (LDI) as well as a reducible chain extender generated from L‐cystine (Cys‐PA) (Scheme S1, Supporting Information). The structure of MPU was confirmed by proton nuclear magnetic resonance (^1^H NMR), Fourier transform infrared (FTIR), and gel permeation chromatography (GPC) analysis (Figures S1–S3, Supporting Information). The MPU prepared could self‐assemble into micelles with diameters around 53 nm and negative surface charges, as determined by dynamic light‐scattering (DLS) and transmission electron microscopy (TEM) (Figures S4 and S5 and Table S1, Supporting Information). The assembled structure of MPU micelles was visually clarified by computational simulation using a dissipative particle dynamics (DPD) model. The result presented a spherical core–shell structure with a hydrophobic core formed by insoluble PCL soft segments and surrounded by an acid‐detachable hydrophilic BPEG corona. The hard segments were located mainly at the subsurface, with some still distributed in the micellar core due to neighboring hydrophobic soft segments (Figures S6 and S7, Supporting Information). The alkyne sites on the interface enabled a post‐conjugation of targeting ligands^[^ ^25^, ^26^, ^27^ ^]^ or shell‐crosslinking^[^ ^14^ ^]^ via click chemistry after the formation of polymer micelles.

To achieve reversible and click crosslinking, we designed and synthesized a reduction‐cleavable crosslinker (SS‐Az) (Schemes S2 and S3, Supporting Information). The obtained crosslinker contains a disulfide linkage and two azide sites, allowing for an efficient crosslinking of MPU micelles using a copper catalyzed alkyne‐azide cycloaddition (CuAAC) in aqueous solution. The success of crosslinking was first verified by ^1^H NMR spectra, where the characteristic peaks of 1,2,3‐triazole ring (8.0 ppm) and the methylene protons near the ring (4.5, 5.5 ppm) were observed for crosslinked MPU micelles (CMPU) (Figure S11, Supporting Information). TEM imaging indicated that the micelles remained their well‐dispersed spherical structures with click reaction at interface (Figure S5, Supporting Information). However, the micellar size increased from 53 to 111 nm after crosslinking (Figure S4, Supporting Information), which may be due to the change of self‐assembled structure or the existence of reaggregation. To understand this phenomenon, we measured the mass‐average molecular weight of micelles using static light scattering (SLS). The calculated aggregation number (*N*_agg_) of CMPU micelles was almost twice as large as that of MPU micelles (Figure S12 and Table S1, Supporting Information). This result implies possible inter‐micelle crosslinking that leads to reaggregation of micelles.

To further prove the success of crosslinking, the MPU and CMPU micelles were treated with 10‐fold volume of *N,N*‐Dimethylformamide (DMF) and analyzed with DLS. It was found that the size of CMPU micelles increased nearly twofold due to the swelling of the hydrophobic segments in the presence of DMF,^[^ ^12^ ^]^ while the structures of MPU micelles were completely disrupted owning to the dissolution of polymers in DMF (Figure S13, Supporting Information). The results indicate that click crosslinking enables CMPU micelles to withstand dissolution in good solvent. The crosslinking of MPU micelles was also confirmed using fluorescence resonance energy transfer (FRET), a facile and powerful tool to detect the molecular interactions within the range of 10 nm and monitor the process and dynamics of self‐assembly in real time.^[^ ^28^, ^29^ ^]^ As a pair of FRET dyes, doxorubicin (DOX, donor) and 3,3′‐diethylthiadicarbocyanine iodide (Cy5, acceptor) were encapsulated into MPU and CMPU micelles separately, followed by mixing the fluorescent‐loaded micelles. As shown in Figure S14A (Supporting Information), with mixing of DOX@MPU and Cy5@MPU micelles, an increase of fluorescence intensity at 695 nm was observed over time, which means that the two dyes were exchanged between the micelles and in a close proximity.^[^ ^29^ ^]^ By contrast, the mixture of DOX@CMPU and Cy5@CMPU did not generate evident FRET signal (Figure S14B, Supporting Information), suggesting an inhibited movement of polymeric chains and enhanced kinetic stability of micelles after crosslinking.

To investigate whether crosslinking improves the thermodynamic stability of micelles, the particle sizes under different dilution times with water or phosphate buffered saline (PBS) were measured by DLS. We found that the diameters and size distributions of MPU micelles increased greatly with water or buffer addition, while those of CMPU were almost unchanged even when diluted more than 700 times (**Figure** **1**A–C and Figures S15 and S16, Supporting Information). Such a stability of CMPU is sufficient for application in the body environment.^[^ ^30^ ^]^ Further, the FRET pair DOX and Cy5 were coloaded in MPU and CMPU micelles, and the fluorescence spectra of micelles upon dilution were collected (Figure S17, Supporting Information). It can be noticed that the FRET efficiency of DOX+Cy5@MPU decreased significantly (Figure 1D), while that of DOX+Cy5@CMPU changed slightly (Figure 1E). On the other hand, once administered into the bloodstream, micelles are immediately mixed with blood cells, plasma proteins, surfactants, and many other components.^[^ ^31^ ^]^ Therefore, they should realize their high stability against the existence of blood components to ensure longevity.^[^ ^32^ ^]^ To this end, the stability of micelles was monitored in the presence of sodium dodecyl sulfate (SDS) surfactant, bovine serum albumin (BSA) protein and fetal bovine serum. As seen in Figure S18 (Supporting Information), the size distributions of MPU micelles changed rapidly under simulated physiological conditions, while those of CMPU micelles remained basically constant after various treatments. These results demonstrate a high stability of MPU micelles after click crosslinking, which is helpful to avoid micellar disassembly and premature drug release in vivo.

**Figure 1:**
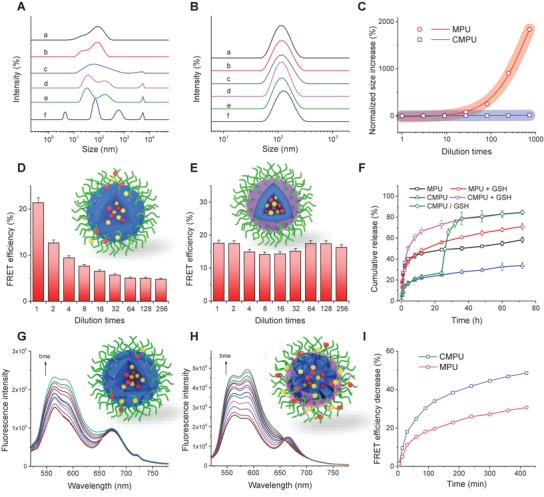
Enhanced stability and promoted redox‐responsivity of crosslinked micelles. A,B) Size distributions of MPU micelles A) and CMPU micelles B) diluted with water for a) 1, b) 3, c) 10, d) 33, e) 100 and f) 333 times. C) Normalized increase in size of MPU micelles before and after crosslinking upon dilution with water. D,E) Normalized change in FRET efficiency of MPU micelles D) and CMPU micelles E) encapsulated with DOX and Cy5 upon dilution with water for different times. The insets in (D,E) show schematic illustrations of DOX+Cy5@MPU and DOX+Cy5@CMPU micelles under dilution, respectively. F) Cumulative release of DOX from MPU and CMPU micelles in phosphate buffered saline (PBS, 10 × 10^−3^ m, pH 7.4) solutions. MPU+GSH and CMPU+GSH indicate release media containing 10 × 10^−3^ m GSH, and CMPU/GSH shows release media with GSH addition at 24 h. G,H) Fluorescence emission spectra (λ_ex_ = 480 nm) of MPU micelles G) and CMPU micelles H) encapsulated with DOX and Cy5 in the presence of 10 × 10^−3^ m GSH for different times. The insets in (G,H) show schematic illustrations of DOX+Cy5@MPU and DOX+Cy5@CMPU micelles after GSH treatment, respectively. I) Normalized decrease in FRET efficiency of DOX+Cy5@MPU and DOX+Cy5@CMPU micelles incubated with 10 × 10^−3^ m GSH for different times.

To verify whether reversible crosslinking of MPU micelles enables controlled release of payloads in tumor microenvironment, a model drug DOX was encapsulated into the micelles followed by a click crosslinking. Evidently, DOX@MPU micelles displayed a typical burst drug release in PBS solution (pH 7.4) (Figure 1F), while the release of DOX was much slower for DOX@CMPU under both neutral and weak acidic conditions (pH 6.5) (Figure S19, Supporting Information). The result verifies a high stability of crosslinked micelles even after detachment of PEG corona. Moreover, a limited acceleration of drug release was achieved in the presence of reducing agent (10 × 10^−3^ m GSH) for uncrosslinked micelles (Figure 1F). This phenomenon is in agreement with our previous findings,^[^ ^25^ ^]^ which may arise from the shielding of disulfide bonds by the soft segments leading to a steric repulsion against the penetration of GSH. Interestingly, DOX@CMPU micelles exhibited a much higher drug release rate than uncrosslinked formulation under 10 × 10^−3^ m of GSH, imparting a sensitive “on‐off” switch for controlled release (Figure 1F). This result seems counterintuitive and contradictory to other studies,^[^ ^33^ ^]^ as it is generally believed that CMPU micelles need to break their crosslinking first for subsequent penetration of GSH into the interior core to attack the disulfide bonds linked with hydrophobic segments. Such a two‐stage degradation of crosslinked polymers is in principle slower than that of uncrosslinked ones. To address this issue, we postulated that the enhanced responsivity might be associated with the change of hierarchical architecture of assemblies during crosslinking, as it has been shown that crosslinkers could chemically induce or kinetically trap the morphological transition of polymeric assemblies.^[^ ^34^ ^]^

To confirm this hypothesis, we performed ^1^H‐^1^H nuclear Overhauser enhancement spectroscopy (NOESY) experiment on the polymers before and after crosslinking. Obviously, a new correlation peak between the crosslinker protons (2.96 ppm) and the methylene next to disulfide bond of Cys‐PA (2.65 ppm) was observed for CMPU (Figure S20, Supporting Information), further confirming the success of click reaction between Cys‐PA and SS‐Az. Moreover, it is interesting to notice that the correlation between PCL (2.32–2.36, 4.08 ppm) and Cys‐PA (2.20, 3.83 ppm) were diminished (**Figure** **2**A,B and Figure S20, Supporting Information), while the NOE signals between PEG protons (3.61, 3.31 ppm) and Cys‐PA (3.36, 3.83 ppm) as well as that among Cys‐PA groups (3.48, 3.83 ppm) were clearly observed after crosslinking (Figure 2C,D). These results reveal a possible migration and aggregation of Cys‐PA moieties from the core to the subsurface layer of micelles due to the azide/alkyne click reaction occurring mainly at the micellar interface, which may induce a reassembly process (CIRA) and microphase separation between the soft and hard segments.

**Figure 2:**
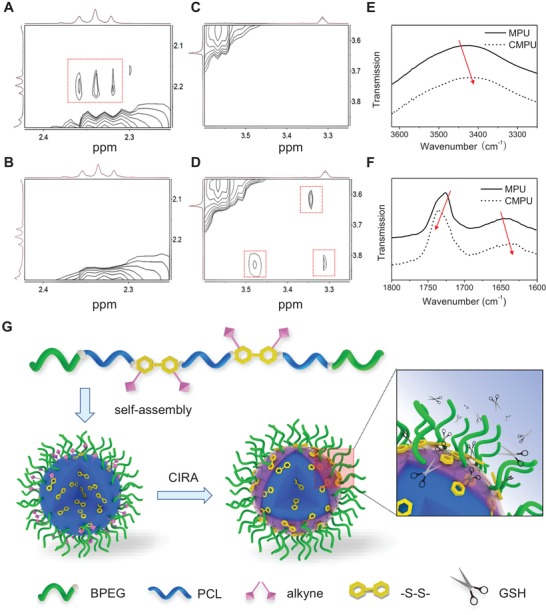
Crosslinking induced reassembly of MPU micelles. ^1^H−^1^H NOESY spectra of MPU micelles in CD_4_O A,C) before and B,D) after cross‐linking. FTIR spectra of lyophilized MPU micelles before and after cross‐linking in the E) N–H and F) C=O stretching regions. G) Schematic representation of CIRA process of multiblock polyurethane micelles.

The CIRA was also supported by FTIR analysis. As shown in Figure 2E, the N−H stretching vibration at 3300–3550 cm^−1^ was shifted to lower frequencies after crosslinking due to an enhanced hydrogen bonding strength.^[^ ^35^ ^]^ More importantly, a blue shift of C=O stretching band centered at 1730 cm^−1^ corresponding to free carbonyl of urethane groups,^[^ ^36^ ^]^ and a red shift of hydrogen‐bonded ordered urea carbonyl around 1640 cm^−1^ were observed (Figure 2F).^[^ ^37^ ^]^ The results verify an enhanced microphase separation between the soft and hard segments and strengthened hydrogen bonding within urea‐bearing hard domains after crosslinking. The aggregation of hard domains can lead to the gathering of disulfide linkages around the micellar interface, thus promoting the reduction responsivity and drug release rate in the presence of GSH (Figure 2G). To further justify the improved responsivity, the micelles co‐loading with DOX and Cy5 were treated with 10 × 10^−3^ m GSH. It was found that the FRET efficiency of DOX+Cy5@MPU declined moderately under reductive environment (Figure 1G,I), while that of DOX+Cy5@CMPU decreased much faster, with an emission at 690 nm quickly disappearing over time (Figure 1H,I). The result agrees well with NOESY, FTIR and drug release experiments, indicating an unusual improvement of redox‐responsivity by crosslinking. To our knowledge, this is the first example of a polymeric assembly with stability and responsivity increased simultaneously by crosslinking, which provides a new strategy to address the dilemma of drug retention and release for controlled delivery.

The promising properties granted by CIRA inspired us to further evaluate the capacity of CMPU for intracellular drug delivery. The MCF‐7 cells were cultured with DOX‐ and Cy5‐coloaded micelles for different times and observed by confocal laser scanning microscope (CLSM). As seen in **Figure** **3**A, there was no FRET fluorescence observed in the cells incubated with DOX+Cy5@MPU, and the distribution of intracellular DOX fluorescence was similar to that of free drugs (Figures S22 and S23, Supporting Information). The result indicates that MPU micelles were dissociated upon contacting with cells, resulting in premature drug leakage outside the cells. In contrast, the cells treated with DOX+Cy5@CMPU showed remarkable FRET signal in the cytoplasm in 1 h, suggesting structural integrity of crosslinked micelles during cellular uptake. After 4 h of incubation, the FRET signal diminished and strong DOX fluorescence was observed inside the nucleus, owning to triggered intracellular release of drugs in response of GSH. It is worth noting that DOX+Cy5@CMPU manifested stronger intracellular fluorescence than DOX+Cy5@MPU in both DOX and Cy5 imaging channels, which may be related to the higher responsivity and faster release property of crosslinked micelles. Besides, the cell entry efficiency of micelles with different stability during uptake should also be taken into consideration.^[^ ^38^ ^]^ With this in mind, we further assessed the mechanism of cell internalization using flow cytometry. As shown in Figure 3B, CMPU micelles showed much higher intracellular fluorescence intensity than MPU micelles even in the presence of buthionine sulphoximine (BSO) that inhibits the production of GSH in cells,^[^ ^39^ ^]^ revealing a greater efficiency of cellular uptake after crosslinking. Moreover, as shown in Figure 3C, the internalization rates of both MPU and CMPU were greatly inhibited at 4 °C, revealing energy‐dependent cellular uptake processes. In particular, the cell entry of DOX@MPU was reduced by chlorpromazine and colchicine, indicating clathrin‐mediated endocytosis and macropinocytosis.^[^ ^40^ ^]^ The uptake of DOX@CMPU could be associated with macropinocytosis due to the lowest internalization rate in the presence of colchicine.^[^ ^41^ ^]^ The different endocytic pathways may account for the enhanced cell internalization of CMPU micelles.^[^ ^42^ ^]^ This phenomenon is of great interest and worth further investigation. The improved cell trafficking and intracellular DOX release would result in higher drug efficacy. However, we found that DOX@CMPU was less effective in killing MCF‐7 tumor cells than MPU formulation (Figure S24, Supporting Information), possibly because the prematurely released DOX from uncrosslinked micelles could rapidly diffuse into the cell nucleus to cause cell death (Figure 3A).^[^ ^43^ ^]^ With this in mind, we used drug‐resistant MCF‐7 cancer cells that can pump free chemotherapeutics out of cells for cytotoxicity assay.^[^ ^44^ ^]^ As expected, DOX@CMPU micelles exhibited much greater therapeutic effect against drug‐resistant tumor cells, with a median inhibitory concentration (IC_50_) 2.5 times lower than that of DOX@MPU (Figure S25, Supporting Information). On the other hand, the drug‐free micelles did not show any toxic effect toward L929 mouse fibroblasts (Figure S26, Supporting Information), suggesting a good cytocompatibility of MPU and CMPUs.

**Figure 3:**
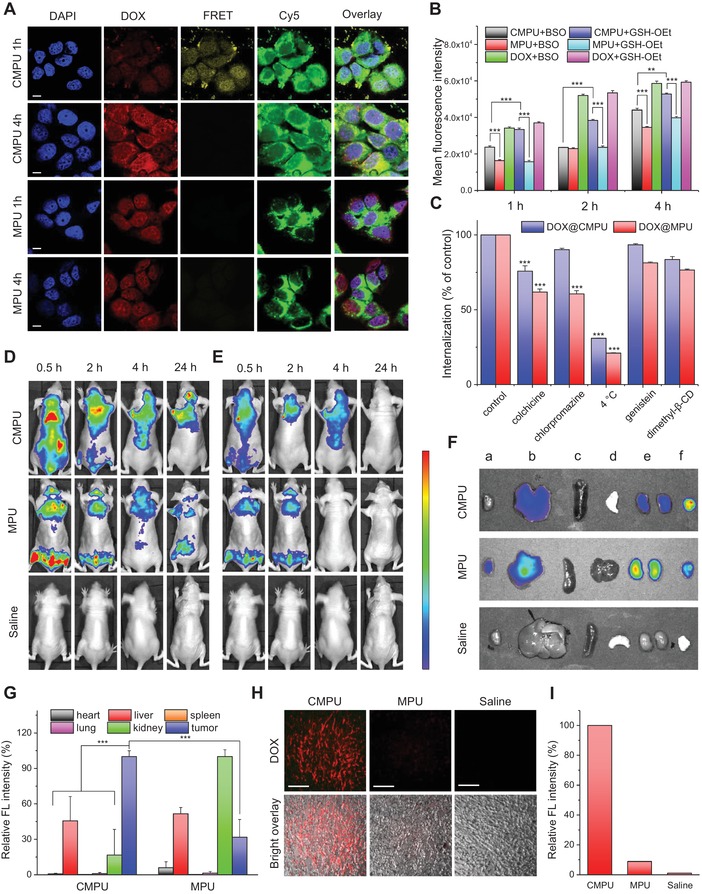
Intracellular drug delivery and in vivo biodistribution. A) CLSM images of MCF‐7 cells incubated with DOX+Cy5@CMPU and DOX+Cy5@MPU for 1 and 4 h. Nuclei of cells were stained with 2‐(4‐amidinophenyl)‐6‐indolecarbamidine dihydrochloride (DAPI, blue). For Cy5 channel, λ_ex_ = 620 nm; for others, λ_ex_ = 480 nm. The scale bars are 10 µm. B) Flow cytometry of BSO‐ or GSH‐OEt‐pretreated MCF‐7 cells incubated with DOX@MPU and DOX@CMPU micelles for 1, 2, and 4 h. C) Flow cytometry of MCF‐7 cells incubated with DOX@MPU and DOX@CMPU micelles for 4 h in the presence of various inhibitors at different temperatures. Cell cultured at 37 °C without inhibitor were set as control. D,E) In vivo imaging of MCF‐7 tumor‐bearing mice at different times after intravenous injection of DOX+Cy5@MPU and DOX+Cy5@CMPU micelles. Mice receiving saline were set as control. D) donor fluorescence channel, λ_ex_ = 480 nm, λ_em_ = 600 nm. E) FRET fluorescence channel, λ_ex_ = 480 nm, λ_em_ = 700 nm. F) The ex vivo imaging of major organs and tumors of nude mice bearing MCF‐7 tumors at 24 h post‐injection of DOX+Cy5@MPU and DOX+Cy5@CMPU micelles (λ_ex_ = 480 nm, λ_em_ = 600 nm), where a,b,c,d,e,f represent heart, liver, spleen, lung, kidney, and tumor, respectively. G) Semi‐quantitative analysis of DOX fluorescence in major organs and tumors. H) CLSM images of tumor tissue slices of nude mice at 24 h post‐injection of DOX+Cy5@MPU, DOX+Cy5@CMPU micelles and saline. The scale bars are 100 µm. I) The fluorescence intensity of tumor slices was quantified and plotted. Statistical significance: (*) *P* < 0.05; (**) *P* < 0.01; (***) *P* < 0.005.

To further explore the benefit of CIRA strategy for in vivo applications, the micelles coloading with DOX and Cy5 were intravenously injected into MCF‐7 tumor‐bearing nude mice via the tail vein, and tracked using an in vivo imaging system. It was found that DOX fluorescence spreaded widely through the abdomen and the FRET effect disappeared within 2 h for DOX+Cy5@MPU group. The result indicates the disassembly of uncrosslinked micelles and premature release of payloads leading to nonspecific biodistribution (Figure 3D,E and Figure S27, Supporting Information). In contrast, CMPU formulation showed both remarkable donor fluorescence and FRET signal rapidly gathering around the tumor tissue, and the FRET emission decreased over time (Figure 3D,E and Figure S27, Supporting Information), suggesting a higher stability, superior targeting capacity and specific intratumor drug release of CMPU micelles granted by CIRA. The ex vivo fluorescent imaging of the anatomized organs of the mouse sacrificed at 24 h evidenced an improved biodistribution of DOX for CMPU formulation, where the fluorescence in tumor was greatly enhanced while those in liver, spleen, and kidney were significantly minimized (Figure 3F and Figure S28, Supporting Information). CLSM imaging of tumor slices further confirmed that the CMPU group showed remarkably stronger DOX fluorescence in tumors than MPU group (Figure 3H,I). The superior targeting effect of CMPU could also be observed in 4T1 tumor‐bearing mice (Figures S29 and S30, Supporting Information).

Next, we evaluated the therapeutic efficacy of MPU and CMPU micelles taking MCF‐7 tumor‐bearing nude mice as a model. As shown in **Figure** **4**A, the tumor volumes in control mice receiving saline administration increased rapidly, while the growth of tumors was remarkably suppressed by the treatment of various DOX formulations. In particular, DOX@CMPU exhibited superior tumor inhibition effect, with mean tumor weight 1.7‐fold and 3‐fold lower than those for DOX@MPU and control groups, respectively (Figure 4B). Furthermore, histological analysis with hematoxylin and eosin (H&E) staining revealed a greater extent of cell remission and necrosis for DOX@CMPU group compared with uncrosslinked micelles and free DOX (Figure 4C). The percentages of apoptotic tumor cells of DOX@CMPU group (≈90%) obtained from nuclear‐associated antigen (Ki‐67) and terminal deoxynucleotidyl transferased dUTP nick end labeling (TUNEL) assays were much higher than those of other groups (Figures S31 and S32, Supporting Information). In addition, although no mice died during the treatment period due to the low dose (Figure S33, Supporting Information), apparent weight loss was detected in mice treated with free DOX (Figure S34, Supporting Information) and, particularly, small amount of cell necrosis of kidney was noticed for mice treated with free DOX and DOX@MPU (Figure S35, Supporting Information). In contrast, no significant decrease in body weights and abnormality of major organs was detected in CMPU formulations (Figures S34 and S35, Supporting Information), demonstrating that CIRA provides an effective strategy for the development of stable and smart nanoplatform for safe and specific drug delivery in vivo. Further work is ongoing to demonstrate the versatility of CIRA strategy using different kinds of polymeric systems, stimuli‐sensitive crosslinkers and other disease models.

**Figure 4:**
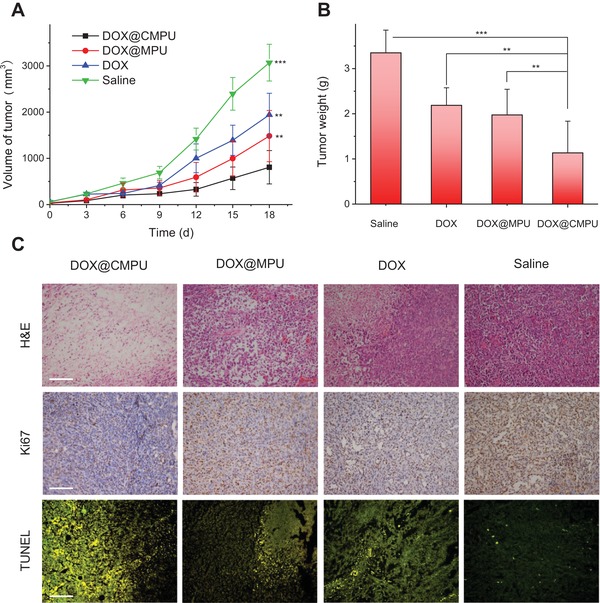
In vivo anticancer efficacy. A) Change of tumor volume after intravenous administration of saline, DOX, DOX@MPU and DOX@CPU micelles in MCF‐7 tumor‐bearing nude mice. B) Mean weights of tumors separated from animals with different treatments. C) Ex vivo histological analyses of tumor sections. H&E staining, Ki67 and TUNEL immunofluorescence staining analyses of tumor sections. In TUNEL analysis, the apoptotic cells were stained green. The scale bars are 100 µm. Statistical significance: (*) *P* < 0.05; (**) *P* < 0.01; (***) *P* < 0.005.

In summary, we have developed a model multiblock polyurethane bearing disulfide linkages in the backbone and clickable active sites in the side chains. The polymer self‐assembled into core–shell micelles in an aqueous solution, and underwent a crosslinking induced reassembly and microphase separation between the soft and hard segments. The CIRA drove the migration of disulfide moieties from the inner core to the subsurface of micelles due to a facile click reaction occurring at the micellar interface. As a result, the thermodynamic stability and redox‐responsivity of micelles could be improved simultaneously, leading to an enhanced tumor targeting, specific intracellular drug delivery and excellent therapeutic efficacy both in vitro and in vivo. Our work provides a new insight into the self‐assembly of macromolecules and a promising nanoplatform for theranostics applications.

## Conflict of Interest

The authors declare no conflict of interest.

## Supporting information

Supporting Information
