# Supplementary material for: Crosslinking Induced Reassembly of Multiblock Polymers: Addressing the Dilemma of Stability and Responsivity
Source: Adv Sci (Weinh). 2020 Mar 6;7(8):1902701. doi: 10.1002/advs.201902701 (PMC7175344; doi:10.1002/advs.201902701)
Supplement: Supplementary file 1 — Supporting Information [file ADVS-7-1902701-s001.pdf]

Copyright WILEY-VCH Verlag GmbH & Co. KGaA, 69469 Weinheim, Germany, 2020.

ADVANCED SCIENCE
Open Access

Supporting Information

for *Adv. Sci.,* DOI: 10.1002/advs.201902701

Crosslinking Induced Reassembly of Multiblock Polymers: Addressing the Dilemma of Stability and Responsivity

Rui Yang, Yi Zheng, Xiaoyu Shuai, Fan Fan, Xueling He, Mingming Ding, Jianshu Li, Hong Tan,* and Qiang Fu*

Copyright WILEY-VCH Verlag GmbH & Co. KGaA, 69469 Weinheim, Germany, 2016.

Supporting Information

Crosslinking Induced Reassembly of Multiblock Polymers: Addressing the Dilemma of Stability and Responsivity

Rui Yang, Yi Zheng, Xiaoyu Shuai, Fan Fan, Xueling He, Mingming Ding, Jianshu Li, Hong Tan*, Qiang Fu*

Materials and Method

Materials

L-lysine ethyl ester diisocyanate (LDI, 97%) was attained from Nantong Dahong Chemical Co., Ltd. (Jiangsu, China). Polycaprolactone (PCL, 99%, MW 2000) was purchased from Dow Chemical Co., Ltd. (Midland, USA). 1-Hydroxybenzotrizole (HOBt), 2-bromoethylamine hydrobromide (98.0%), 3,3'-dithiodipropionic acid (DTDPA) and bovine serum albumin (BSA) were purchased from Adamas Reagent, Ltd. (Shanghai, China). Sodium ascorbate (99.0%) was purchased from Aladdin Reagent Database Inc. (Shanghai, China). Sodium azide was obtained from Sanland-chem, International Inc. (Xiamen, China). 3,3'-Diethylthiadicarbocyanine iodide (Cy5) was obtained from Alfa Aesar (China) Chemistry Co., Ltd. (Shanghai, China). *N,N'*-Dicyclohexylcarbodiimide (DCC, ≥98.0%), copper (II) sulfate pentahydrate ($CuSO_4•5H_2O$), dichloromethane (DCM), 1,2-dichloroethane, diethyl ether, ethyl acetate, methanol, hydrogen chloride, sodium chloride, triethylamine, isopropanol, sodium bicarbonate ($NaHCO_3$), tetrahydrofuran (THF), sodium dodecyl sulfate (SDS), *N,N*-dimethylformamide (DMF), *N,N*-dimethylacetamide (DMAc), anhydrous sodium sulfate (Na_2SO_4), sodium hydroxide (NaOH) and acetone were provided by Chengdu Kelong Chemical Reagent Company (Sichuan, China). Glutathione (GSH) was purchased from Biofroxx (Einhausen, German). Doxorubicin hydrochloride (DOX· HCl) was purchased form

Dalian Meilun Biotechnology Co., LTD. (Dalian, China). 3-(4,5-dimethyl-2-thiazolyl)-2,5-diphenyl-2-H-tetrazolium bromide (MTT), L-buthionine-sulfoximine (BSO), glutathione ethyl ester (GSH-OEt), and 4',6-diamidino-2-phenylindole (DAPI) were purchased from Sigma (St. Louis, MO, USA). pH-sensitive methoxyl-poly(ethylene glycol) with a benzoic-imine linkage (BPEG) and L-cystine-derivatized diamine chain extender containing redox-responsive disulfide bond and clickable alkynyl groups (Cys-PA) were synthesized according to a previous report.[1]

Characterization

All Nuclear magnetic hydrogen spectroscopy (^{1}H NMR) and nuclear magnetic carbon spectroscopy (^{13}C NMR) were recorded at room temperature on a Bruker Avance III HD 400MHz spectrometer using and $CDCl_3$ or DMSO-d_6 as the solvents and tetramethylsilane (TMS) as an internal standard.

2D nuclear Overhauser effect spectroscopy (NOESY) ^{1}H NMR spectra were measured using an AVANCE III HD spectrometer (400 MHz, JEOL) with a sweep width of 4000 Hz into 1024 data points. The relaxation delay was 2 s and the mixing time was 0.3 s. The number of scans was 4.

Fourier transform infrared (FTIR) spectra were recorded from a Nicolet iS10 spectrometer (Thermo Electron Corporation, U.S.A) from 4000 to 600 cm^{-1} by a transmission mode. The polymer was dissolved in chloroform (5%) and dropped onto potassium bromide tablets. Then the potassium bromide tables were dried at 60 °C for 4 h, and dried vacuum drier at 40 °C for 4 h.

Mass spectra (MS) were acquired using an HP1100-LC/MSD (Agilent Technologies, Santa Clara, CA, USA) with atmosphere pressure chemical ionization (positive mode).

The molecule weight and molecular weight distribution were determined by gel permeation chromatography (GPC) with an HLC-8320 (TOSOH Corporation, Japan) at room temperature using THF as an eluent. The molecular weights were calibrated against polystyrene (PS, MW

47200) standards. The sample concentration was 2 mg mL^{-1} and the flow rate was 1.0 mL min^{-1}.

The morphology of samples was observed using a transmission electron microscope (TEM), which was acquired on a model H-600-4 (Hitachi, Ltd., Japan) operated at an accelerating voltage of 75 KV. TEM grids were prepared by depositing a diluted suspension of sample onto a copper grid with staining with 1% (w/v) phosphotungstic acid for 3 min, the excessive solution was blotted away and air dried before imaging.

Fluorescence measurement was conducted on an F-4600 FL spectrophotometer (Hitachi, Ltd., Japan). For pyrene fluorescence, the excitation spectra were collected from 206 nm to 406 nm at an emission wavelength (λ_{em}) of 372 nm, the emission spectra were collected from 350 nm to 550 nm at an excitation wavelength (λ_{ex}) of 331 nm. For Forster resonance energy transfer (FRET) measurements, the emission spectra were collected from 500 nm to 800 nm at a λ_{ex} of 480 nm.

The size and zeta potential of polymer assemblies were obtained on a Zetasizer Nano ZS instrument (Malvern Instruments Ltd., UK) at room temperature at an angle of 90°. The relevant data were presented as mean ± standard deviation (SD) based on triplicate independent experiments.

Synthesis of clickable multiblock polyurethane (MPU)

The multiblock polyurethane was synthesized from PCL, BPEG, LDI and Cys-PA according to our previous reports.[1, 2] Briefly, PCL (3.2 g) dissolved in DMAc was first copolymerized with LDI (0.949 g) at 60 °C under a dry nitrogen atmosphere in the presence of stannous octoate catalyst for 1 h. After cooling to room temperature, chain extender Cys-PA (0.628 g) was added and allowed to react with prepolymers for 1 h at room temperature, followed by another 2 h at 60 °C. Finally, BPEG (1.674 g) was added to react for 6 h. The polymer obtained was precipitated in anhydrous ethyl ether and dried under vacuum at 60 °C for 3 d.

The chemical structure of MPU was characterized by ^{1}H NMR, FTIR and GPC. As shown in Figure S1, all characteristic peaks of PCL, PEG, and LDI can be found. The peaks at 3.97 (-COOC***H***$_2$-), 2.26 (-C***H***$_2$COO-),1.52 (-C***H***$_2$CH$_2$C***H***$_2$-), and 1.30 ppm (-CH$_2$C***H***$_2$CH$_2$-) are assigned to the methylene protons of PCL unit. The sharp peak at 3.50 ppm is attributed to the methylene protons of PEG block (-C***H***$_2$C***H***$_2$O-). The chemical shifts of methylene (-C***H***$_2$-OCO-) and methyl protons (-C***H***$_3$) in the ethoxyl group of LDI are at 4.07 and 1.15 ppm, respectively. The characteristic peak of the imine proton (-***H***C=N-) is at 8.20 ppm, and the signals at 8.02 and 7.89 ppm are originated from benzene ring of BPEG. In addition, peaks at 2.73 and 3.07−3.16 ppm are ascribed to the alkynyl proton (-C≡C***H***) and methylene protons next to the disulfide bond (-S−S−C***H***$_2$-) in Cys-PA, respectively, demonstrating that the multifunctional chain extender has been successfully introduced into the chains of polyurethanes. The FTIR spectra of polyurethanes are shown in Figure S2. The stretching band in the 1600−1800 cm^{-1} region is overlapped by the absorption of ester carbonyl groups of PCL and free and hydrogen-bonded carbonyl of urethane groups, where a shoulder observed at 1654 cm^{-1} is ascribed to the hydrogen-bonded carbonyl of urea groups. A broad stretching band around 3340 cm^{-1} is mainly attributed to the hydrogen-bonded N-H stretching vibration. GPC analysis indicates that the weight average molecular weight of MPU is about 70933 g mol^{-1}, with monodisperse and quite narrow molecular weight distributions (PDI 1.20, Figure S3). All the above results prove that the polymer has been successfully synthesized.

Synthesis of azidoethylamine (AzEA)

AzEA was synthesized according to Scheme S2. In brief, 2-bromoethylamine hydrobromide (10.25 g) was dissolved in distilled water with mild stirring. Sodium azide (9.75 g) was added carefully and the mixture was kept at 70 °C with refluxing for 12 h. Thereafter, the system was cooled to 0 °C and NaOH (7.00 g) was added. The product was extracted with anhydrous ethyl ether and dried over Na_2SO_4. After filtration of the solution, the solvent was allowed to evaporate under atmospheric conditions (yield: 50−60%).^{1}H NMR

(400 MHz, DMSO-d_6, TMS, δ in ppm): 1.53 (s, 2H, -NH_2), 2.89 (t, 2H, -CH_2-NH_2), 3.45 (t, 2H, -CH_2-N_3).FTIR (cm^{-1}): 3357.00 (s, v N−H), 2931.63 (s, v CH_2), 2102.35 (s, v -$N^+{\equiv}N$), 1584.00 (s, δ NH_2), 1473.81 (s, δ CH_2).

Synthesis of reduction-cleavable crosslinker (SS-Az)

The reduction-cleavable crosslinker (SS-Az) crosslinker was synthesized from AzEA and DTDPA (Scheme S3). Briefly, DTDPA was completely dissolved in DCM precooled in an ice water bath, then AzEA, DCC and HOBt were added in turn to the solution and stirred for 1 h. The reaction was kept at room temperature for 24 h. Then the solvent was evaporated under atmospheric conditions. Afterward, 100 mL 0.5 M hydrochloric acid was added, and product was extracted with ethyl acetate. The organic phase was collected and washed with saturated solution of $NaHCO_3$, sodium chloride and distilled water, and dried over anhydrous Na_2SO_4 overnight. Then the solution was filtered and condensed under reduced pressure. The crude product was purified by recrystallization for three times in DCM, and dried under vacuum at 45 °C for 3 d (yield: 72%).

^{1}H NMR (400 MHz, $CDCl_3$, TMS, δ in ppm): 2.63 (t, 2H, -CH_2-N_3), 2.89 (t, 2H, -CH_2-CO), 3.47 (s, 4H, -CH_2-NH, CH_2-S-S), 6.3 (s, 1H, NH-CO). ^{13}C NMR (400 MHz, $CDCl_3$, δ): 33.8, 35.6,38.9, 50.6, 171.1. FTIR (cm^{-1}): 3295.4 (s, v -NH),2127.7 (s, v -N_3), 1646 (s, v -C=O).

Self-assembly of multiblock polyurethane

The assemblies of block copolymers were prepared using a dialysis method. Briefly, a solution of MPU (25 mg) in 2.5 mL of DMAc was added dropwise into 25 mL of deionized water with quickly stirring. Then the solutions were transferred into a dialysis bag (MWCO 3500) and dialyzed against deionized water for 3 d, changing the external water once 3 h. Finally, the solution was centrifugalized for 15 min at 3000 r min^{-1} and filtered through a 0.45 μm pore-sized syringe filter (Millipore, Carrigtwohill, Co. Cork, Ireland).

Computational simulation

To investigate the structure of MPU micelles, computational simulation was carried out using a dissipative particle dynamics (DPD) model. DPD simulation is a particle-based mesoscopic simulation technique originally introduced by Hoogerbrugge and Koelman in 1992,[3, 4] and further modified by Español and Warren.[5] It has been established as a powerful tool to investigate the self-assembly of amphiphilic copolymers.[6] In our study, we consider an aqueous solution (*W*) of MPU. The system comprises clickable multiblock polymers and water in a cubic box of size 20 × 20 × 20 r_c^3 with periodic boundary condition. MPU was divided into six types of beads (E, A, B, L, C, and S). Simple coarse-grained models of these components are shown in Fig. S6. The calculated interaction parameters in polymeric micellar systems at 298 K are given in Table S2. Detailed simulation methods and equations can be found in our previous reports.[7-11] The DPD simulations were conducted using Materials Studio 5.0 software (Accelrys) installed on a DELL PowerEdge SC430 server. The total beads were 24,000, the spring constant C was chosen as 4.0 and the time step was taken as 0.05. According to Figure S7A, 100,000 DPD steps adopted were sufficient for achieving simulation equilibrium and steady results.

The simulation results were depicted in Figure S7. As shown in the density profiles (Figure S7B), front view (Figure S7C) and cross-sectional view of MPU micelles (Figure S7D), the multiblock polyurethane self-assembles into a spherical core-shell structure with a hydrophobic core formed by insoluble PCL soft segments (blue) and surrounded by a hydrophilic BPEG corona (green). The hard segments composed by Cys-PA (red) and LDI residues (yellow) are located mainly at the subsurface, with some still distributed in the micellar core due to neighboring hydrophobic soft segments.

Crosslink of multiblock polyurethane assemblies

The obtained crosslinker contains a disulfide linkage and two azide sites, allowing for an efficient crosslinking of MPU micelles *via* a copper catalyzed alkyne-azide cycloaddition (CuAAC) in aqueous solution. The degree of crosslinking could be controlled by the feed

ratio of crosslinker. Briefly, MPU micelles (25 mL) were mixed with SS-Az (0.5 and 10 eq) in the presence of sodium ascorbate (30 mg) and $CuSO_4 \cdot 5H_2O$ (20 mg) and incubated at room temperature under moderate stirring for 24 h. Afterward, the solutions were transferred to dialysis tubes (MWCO 3500) and dialyzed against deionized water for 2 d to remove the unreacted SS-Az and traces of the catalyst. Then the crosslinked assemblies (CMPU) solutions were centrifugalized at 3000 r min^{-1} for 20 min and passed through a 0.45 µm pore-sized syringe filter.

To verify the structure of CMPU, the crosslinked assemblies after lyophilization were characterized by ^1H NMR. As shown in Figure S11 and S12, three new peaks appear at 4.5 ppm (CO-NH-C$\boldsymbol{H}_2$), 5.5 ppm ($CH_3C\boldsymbol{H}_2$- triazole) and 8.0 ppm (triazole), which are attributed to the proton of methylene near 1,2,3-triazole and 1,2,3-triazole resulted from CuAAC click reaction. The characteristic peaks of methylene protons near the disulfide bond could also be found at 2.79 (C$\boldsymbol{H}_2$-S-S), 3.55 (C$\boldsymbol{H}_2$-S-S) and 3.91 ppm (CH_2-C$\boldsymbol{H}_2$-S-S). In addition, the characteristic peak of methylene proton near alkynyl at 3.22 ppm (C$\boldsymbol{H}_2$-C≡CH) disappears. The above results verify the successful crosslinking of MPU micelles *via* click chemistry.

Determination of aggregation number (N_{agg})

The weight-average molecular weight of multiblock polyurethane assemblies before and after crosslink were measured by SLS using the Debye plot. The aggregation numbers of MPU and CMPU assemblies were calculated by eq S1:

$$\text{Aggregation Number} = \frac{M_{w,aggregate}}{M_{w,block\ copoolymer}} \quad \text{(S1)}$$

where $M_{w,\ aggregate}$ is the weight-average molecular weight of polymeric assemblies estimated by SLS and $M_{w,\ block\ copolymer}$ is the sum of weight-average molecular weight of polymer obtained by GPC analysis.

DOX and Cy5 encapsulation

To load DOX or Cy5, 1 mL of DOX (1 mg mL^{-1}) or Cy5 (1 mg mL^{-1}) solutions in DCM was added into a bottle, and dried with a flow dry argon. Then, 10 mL of polymer dispersions in water (3mg mL^{-1}) were added into the bottle and ultrasonated for 2 h. The solution was transferred into a dialysis bag (MWCO 3500) and dialyzed against water for 24 h, changing the water every 3 h. The fluorescent dye-loaded assemblies (DOX@MPU or Cy5@MPU) were crosslinked as described above to prepare dye-labeled crosslinked micelles (DOX@CMPU or Cy5@CMPU). Finally, all the solutions were centrifugalized for 10 min at 3000 r min^{-1} and filtered through a 0.45 μm pore-sized syringe filter (Millipore, Carrigtwohill, Co. Cork, Ireland).

To co-encapsulate DOX and Cy5, 1 mL of DOX (1 mg mL^{-1}) solutions in DCM was added into a bottle, and dried with a flow dry argon. Then 10 mL of Cy5@MPU solution were added into the bottle and ultrasonated for 2 h. The solution was transferred into a dialysis bag (MWCO 3500) and dialyzed against water for 24 h, changing the water every 3 h. The DOX and Cy5 co-loaded micelles (DOX+Cy5@MPU) were crosslinked as described above to prepare crosslinked FRET micelles (DOX+Cy5@CMPU). Finally, the solutions were centrifugalized for 10 min at 3000 r min^{-1} and filtered through a 0.45 μm pore-sized syringe filter (Millipore, Carrigtwohill, Co. Cork, Ireland).

DMF dilution test

Aqueous solution of micelles (100 μL) was placed in a vial, followed by the addition of pure DMF (1 mL). The solution was shaken for 1 min to fully mix DMF and water. Then the size and size distribution of micelles were measured by DLS.

Kinetic study of dye-encapsulated micelles

The crosslinking of MPU micelles was proved using fluorescence resonance energy transfer (FRET), which is a facile and straightforward tool to detect the molecular interactions within the range of 10 nm and monitor the process and dynamics of self-assembly in real time.[12, 13] As a pair of FRET dyes, DOX (donor) and Cy5 (acceptor) were encapsulated into

MPU and CMPU assemblies separately as described above. To determine the kinetic stability of MPU and CMPU micelles, the polymeric assemblies encapsulating DOX or Cy5 were mixed for different times and determined with an F-4600 FL spectrophotometer (Hitachi, Ltd., Japan). The donor (DOX) was excited at 480 nm and the emission spectra were recorded at all wavelengths simultaneously.

Stability of multiblock polyurethane micelles against dilution

To investigate whether crosslinking improves the thermodynamic stability of micelles, the multiblock polyurethane micelles before and after crosslinking were diluted with deionized water and phosphate buffer solution (PBS). The particle sizes under different dilution times were measured with a Zetasizer Nano ZS instrument (Malvern Instruments Ltd., UK) at room temperature at an angle of 90°.

In addition, FRET measurements were also conducted to study the stability of assemblies before and after crosslink. In brief, the fluorescence spectra of DOX and Cy5 co-loaded assemblies before and after crosslink upon dilution was collected. The FRET efficiency was calculated from the intensity ratio $I_A/(I_D + I_A)$, where I_A and I_D were the fluorescence intensities at 690 and 550 nm, respectively.

Stability of multiblock polyurethane micelles against surfactant, protein and serum

To investigate the potential stability of multiblock polyurethane micelles under physiological conditions, the MPU and CMPU micelles were incubated with sodium dodecyl sulfate (SDS, 10mg mL^{-1}), bovine serum albumin (BSA, 45 mg mL^{-1}) or fetal bovine serum (20%) with shaking. The size and size distribution of micelles were monitored over time by Zetasizer Nano ZS instrument (Malvern Instruments Ltd., UK).

Responsiveness of multiblock polyurethane micelles

To evaluate the reduction responsivity of MPU assemblies before and after crosslink, DOX+Cy5@MPU and DOX+Cy5@CMPU micelles were treated with 10 mM of GSH and measured with an F-4600 FL spectrophotometer (Hitachi, Ltd., Japan) at different time points.

The donor (DOX) was excited at 480 nm and the emission spectra were recorded at all wavelengths simultaneously. The ratio of fluorescence intensity at 594 nm to that at 670 nm was normalized and plotted agaist time.

Triggered release of DOX

To verify whether reversible crosslinking of MPU assemblies enables controlled release of payloads in tumor microenvironment, a model drug DOX was encapsulated into the micelles and crosslinked as described above. The release of DOX was evaluated using a dialysis method. Briefly, 3 mL of DOX-loaded assemblies before and after crosslink were added into dialysis bags (MWCO 3500) and incubated in 10 mM phosphate buffer solution (PBS, pH 7.4) under constant shaking (100 r min^{-1}) at 37 °C. The release media contain GSH (10 mM) or acid (pH 6.5) to accelerate drug release, tacking PBS solution as a control. To demonstrate the on-off switched release property of crosslinked micelles, 3 mL of DOX@CMPU micelles was first incubated with 10 mM PBS (pH, 7.4), then 10 mM of GSH was added at 24 h. For all the tested groups, 2 mL of release media was sampled and replenished with an equal volume of fresh media at desired time intervals. The amount of DOX released was determined by a UV-vis spectrometer (UV-2600, Shanghai Techcomp Instrument Co., Ltd, Shanghai, China). The release experiments were conducted in triplicate.

Cell internalization

The cellular uptake of DOX and Cy5-coloaded polymeric micelles before and after crosslinking was determined by confocal laser scanning microscope (CLSM) and flow cytometry. For CLSM, MCF-7 breast cancer cells obtained from West China Hospital were seeded in a six-well plate (a coverslip was placed in every well before use) at a density of 1 × 10^5 cells per well and cultured overnight. Then DOX+Cy5@MPU and DOX+Cy5@CMPU assemblies were added separately into the plate with a consistent drug concentration of 10 μg mL^{-1} and incubated for 4 h. Next, the medium was removed and the plate was washed with PBS for three times. Then the cells were fixed with 4% formaldehyde for 30 min and stained

with DAPI for 10 min. At last, the coverslips were mounted with 50% glycerol solution and observed on a CLSM (Olympus FV1000, Japan). For flow cytometry, MCF-7 breast cancer cells were seeded in a six-well plate (a coverslip was placed in every well before use) at a density of 1 × 10^5 cells per well and cultured overnight. The cells were then treated with 10 mM glutathione ethyl ester (GSH-OEt) for 2 h or 0.1 mM L-buthionine-sulfoximine (BSO) for 2 h. Thereafter, DOX+Cy5@MPU and DOX+Cy5@CMPU micelles were added at a consistent drug concentration of 10 μg mL^{-1}, and the cells were incubated for another 4 h. After removing the medium, the plate was washed with PBS for three times. Then the cells were digested by trypsin, centrifuged, and re-suspended in 0.5 mL PBS for flow cytometer measurement (Beckman Cytoflex, USA).

Endocytosis mechanism

To evaluate the endocytosis mechanism of multiblock polyurethane micelles before and after crosslinking, MCF-7 cells were seeded in a six-well plate (a coverslip was placed in every well ahead of use) at a density of 1 × 10^5 cells per well and cultured overnight. Then the cells were pre-incubated with different inhibitors: M-*β*-cyclodextrin (2.5 mM), chloropromazine (10 μg mL^{-1}), colchicine (8 μg mL^{-1}), genistein (50 μg mL^{-1}) for 2 h at 37 °C. Meanwhile, another two groups of cells were pre-incubated without inhibitor at 4 ℃ and 37 °C for 2 h. Cells without pretreatment were set as control. Then DOX@MPU and DOX@CMPU micelles were added into the plate with a consistent drug concentration of 10 μg mL^{-1} and incubated at 37 °C or 4 ℃ for 4 h. Finally, the cells were washed, digested, centrifuged and resuspended for flow cytometer measurement.

MTT assay

MTT assay was performed to evaluate the cytotoxicity of empty MPU or CMPU assemblies against L929 mouse fibroblasts and DOX-loaded formulations against both drug-sensitive and drug-resistant MCF-7 cancer cells. All the cell lines were obtained from West China Hospital. Briefly, L929 mouse fibroblasts or MCF-7 cancer cells were seeded in 96-

well plates at a density of 5× 10^3 cells per well and cultured overnight. Then the L929 fibroblasts cells and MCF-7 tumor cells were treated with drug-free multiblock polyurethane assemblies and DOX-loaded micellar formulations, respectively, with different concentrations. The cells were incubated for 48 h, followed by the addition of 20 μL MTT solution (5mg mL^{-1}) for another 2 h of incubation. Finally, the solution in each well was replaced by 200 μL of DMSO. After shaking the plates for 10 min to dissolve the formazan crystals, the absorption intensity at 490 nm was recorded on a microplate reader (DNM-9602, Nanjing Perlove Medical Equipment Co., Ltd., China).

Construction of tumor model

Five to six-week-old female BALB/c nude mice or KM mice were purchased from Vital River Company in Beijing. All experimental procedures were in accordance with the guidelines for laboratory animals established by the Laboratory Animal Center of Sichuan University. MCF-7 or 4T1 cells were large-scale expanded *ex vivo* in culture medium and collected in PBS. 100 μL of cell suspensions (2.0×10^7 cells mL^{-1}) were injected into the right armpit of BABL/c nude mice or KM mice. The body weight and tumor size were measured every three days. The tumor volume was calculated using the equation $V = ab^2/2$, where “*a*” and “*b*” represent the length and width of tumors, respectively.

In vivo and ex vivo imaging study

To investigate the targeting property and biodistribution of multiblock polyurethane micelles *in vivo*, MCF-7 tumor-bearing nude mice or 4T1 tumor-bearing KM mice were randomly divided into three groups. When the tumors had grown to around 100 mm^3, the mice were intravenously injected with DOX+Cy5@MPU and DOX+Cy5@CMPU micelles *via* the tail vein, and tracked by an IVIS imaging system (Caliper Life Sciences, USA) at different time points. The excitation filter is 490 nm, and the emission filter are 600 and 700 nm. The animals were sacrificed at 24 h post-administration, and tumor tissues and major organs including heart, liver, spleen, lung, and kidney were collected for *ex vivo* fluorescence

examination using the same imaging system. For the confocal analysis, excised tumors were frozen in optimum cutting temperature (OCT) (Sakura Finetek, USA) at -80 °C. The corresponding slices (6 μm) were prepared. The red fluorescence emitted from DOX was collected on a CLSM (Olympus FV1000, Japan).

Antitumor treatment

MCF-7 tumor-bearing nude mice were divided into four groups (five mice per group): saline, DOX@MPU, DOX@CMPU and DOX. When the tumors had grown to 30-50 mm^3, the nude mice were treated with DOX@MPU and DOX@CMPU *via* tail vein every 3 d for 15 d at a DOX dose of 5 mg kg^{-1}. Mice injected with free DOX and saline were set as positive and negative controls, respectively. The tumor sizes were recorded every three days using a digital caliper. On the day of 15, all the mice were sacrificed and the tumors were excised and weighed, the major organs (heart, liver, spleen, lung and kidney) and tumors were collected for further analysis.

The biological safety *in vivo* and tumor suppression effect after the treatment were assessed by hematoxylin-eosin (H&E) staining, terminal deoxynucleotidyl transferase-mediated deoxyuridine triphosphate nick end labeling (TUNEL) assay and nuclear-associated antigen (Ki-67) immunohistochemistry analysis. The tumors and main organs such as liver, kidney, heart, lung, and spleen were collected, embedded with paraffin, and cut into 5-μm-thick sections. The tissues were then stained with H&E, TUNEL, Ki67 and observed with fluorescence microscopy to assess the histopathology alterations.

Statistical analysis

The quantitative data obtained were expressed as means ± standard deviations (SD). Statistical analysis was carried out using the Statistical Package for the Social Sciences (IBM SPSS Statistics software, Version 19, IBM, New York, USA). Student’s t-test or one-way analysis of variance (ANOVA) was performed to determine the statistical significance within the data at 95% confidence levels ($P < 0.05$).

Supporting Figures and Tables

Scheme S1. Synthesis of multiblock polyurethane (MPU)

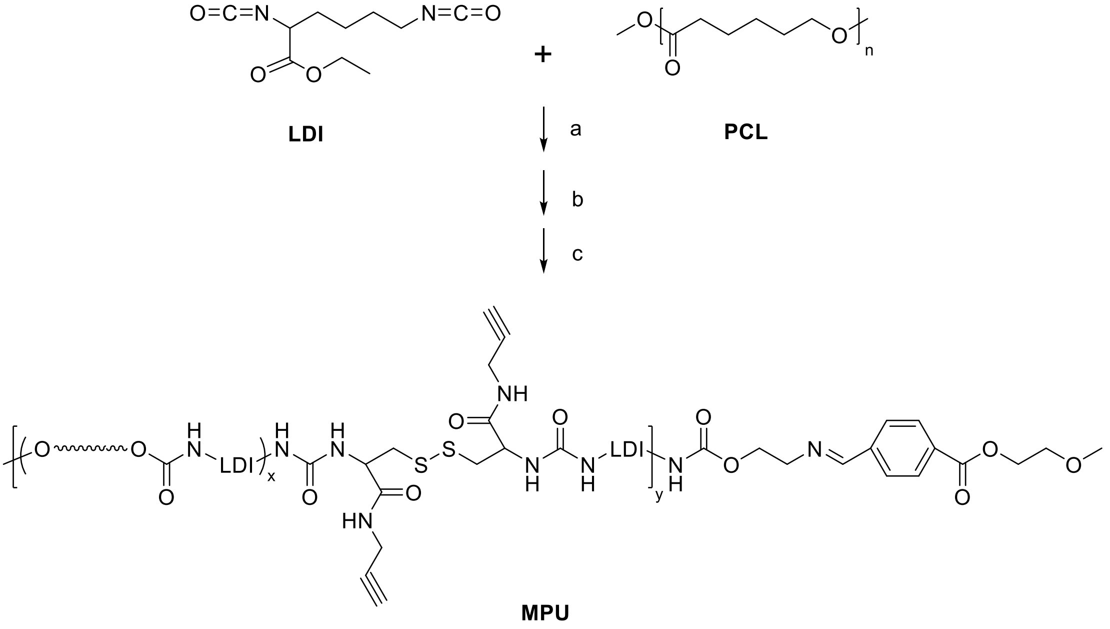

Reagents and conditions: (a) stannous octanoate, DMAc, 60 ℃, 1 h; (b) Cys-PA, DMAc, r.t., 1 h, 60 °C, 2 h; (c) BPEG, DMAc, 60 °C, 6 h.

Scheme S2. Synthesis of azidoethylamine (AzEA)

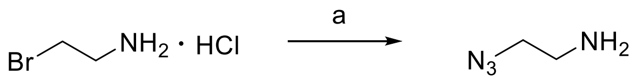

1

Reagents and conditions: (a) sodium azide, distilled water, 70 °C, 12 h (60% yield).

Scheme S3. Synthesis of reduction-cleavable crosslinker (SS-Az)

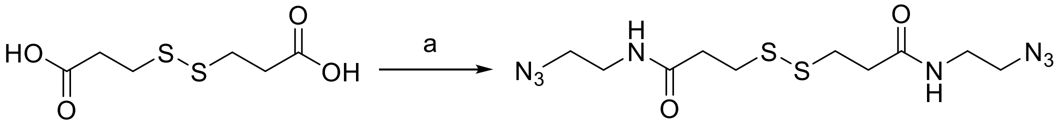

2

Reagents and conditions: (a) DTDPA, DCC, HOBt, DCM, r.t., 24 h (72%).

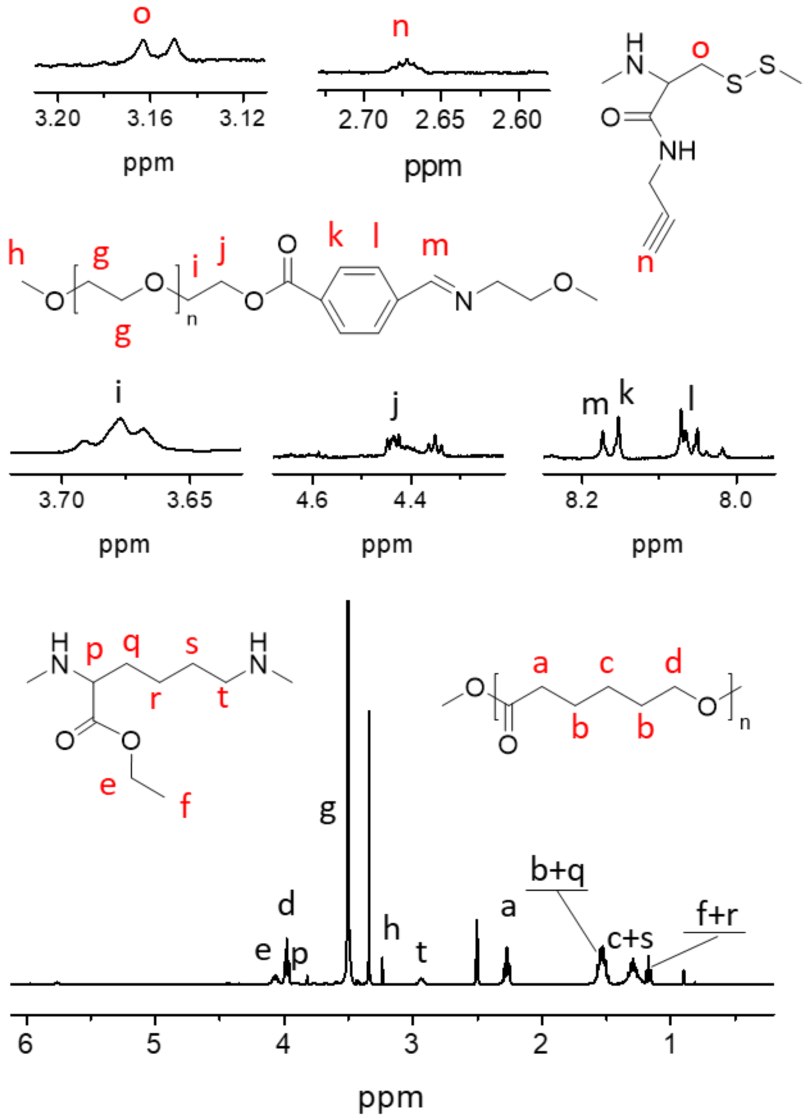

Figure S1. 400 MHz ^{1}H NMR spectra of MPU in DMSO-d_6.

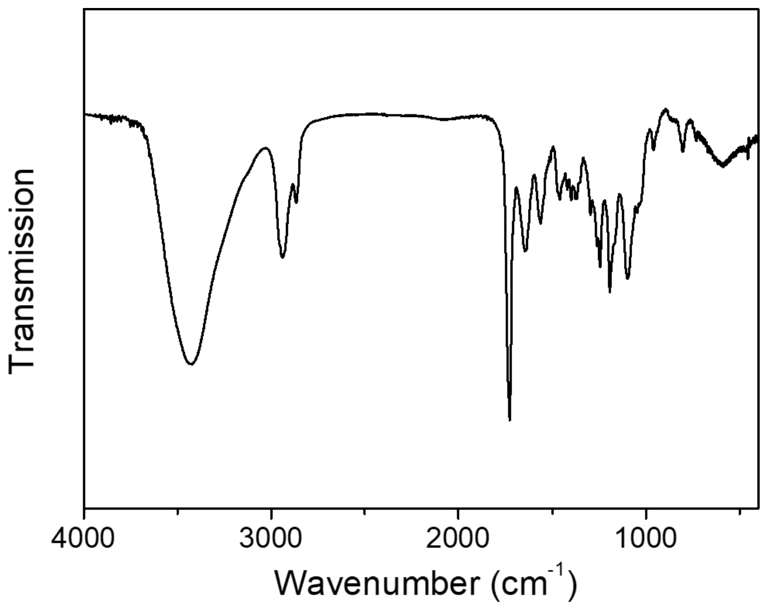

Figure S2. FTIR spectrum of MPU.

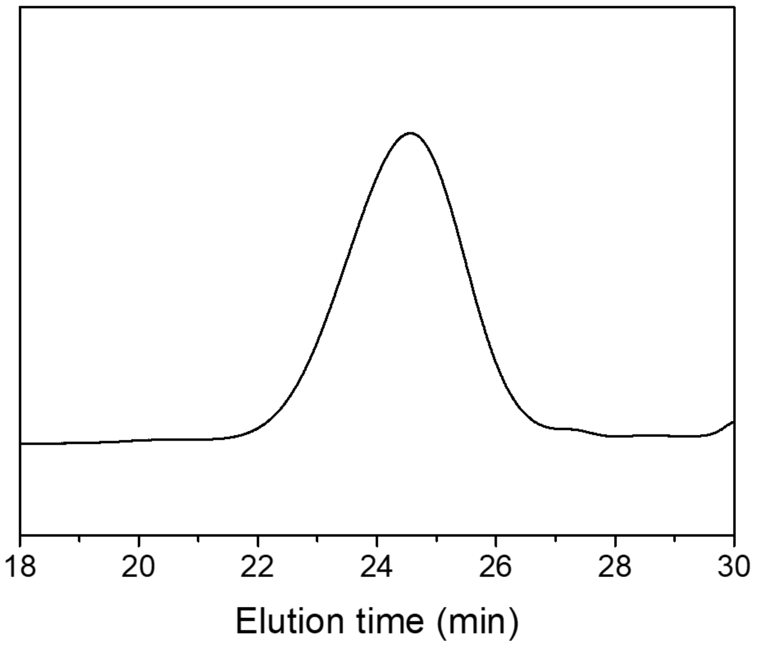

Figure. S3 GPC diagram of MPU.

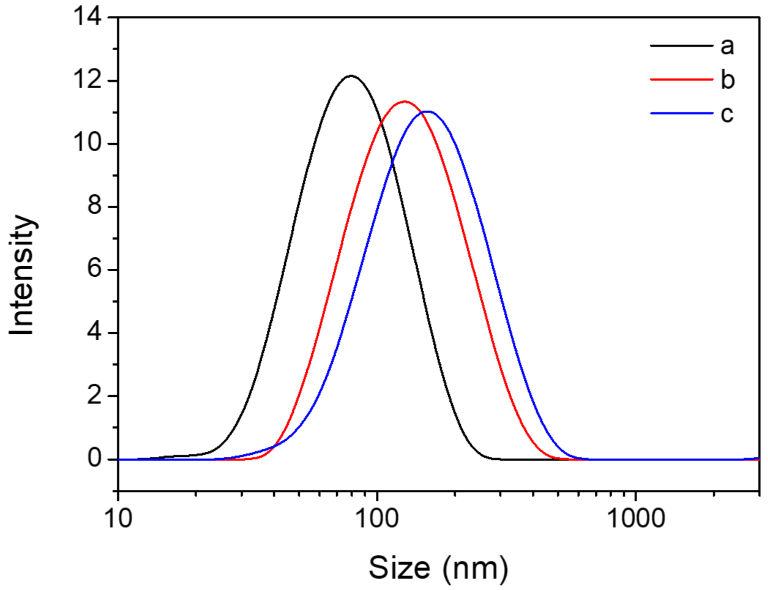

Figure S4. Size distributions of MPU micelles before (a) and after crosslinking with SS-Az feed ratios of 0.5 eq (b) and 10 eq (c).

A
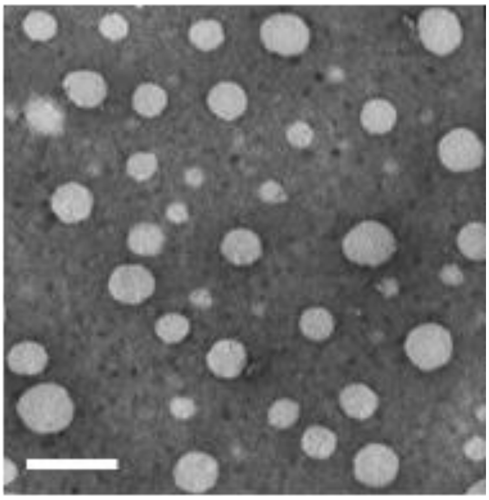

B
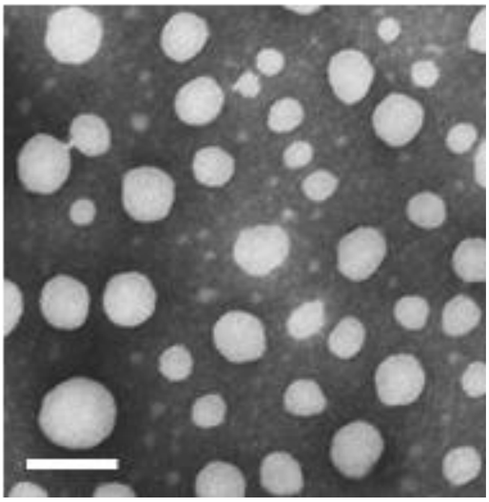

Figure S5. TEM images of MPU micelles before (A) and after (B) crosslink. The scale bar is 100 nm.

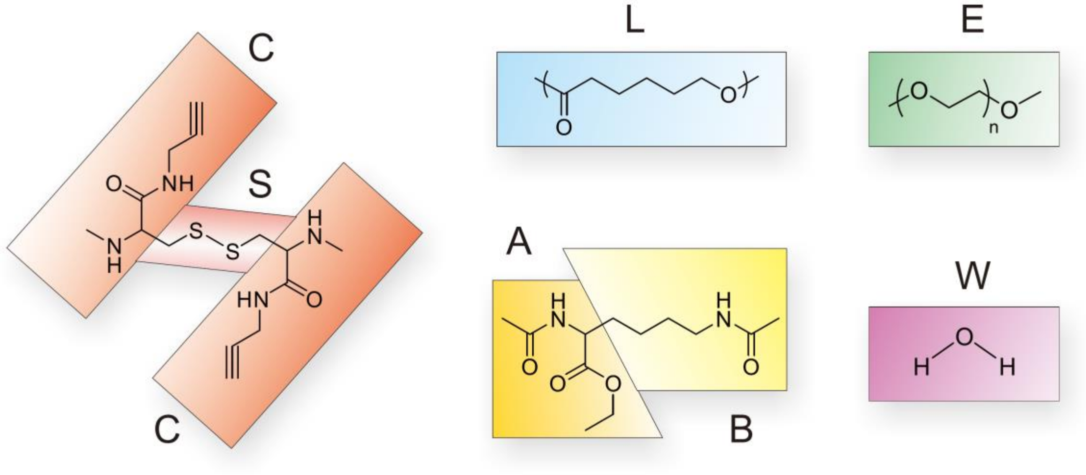

Figure S6. Coarse grained models of multiblock polyurethanes.

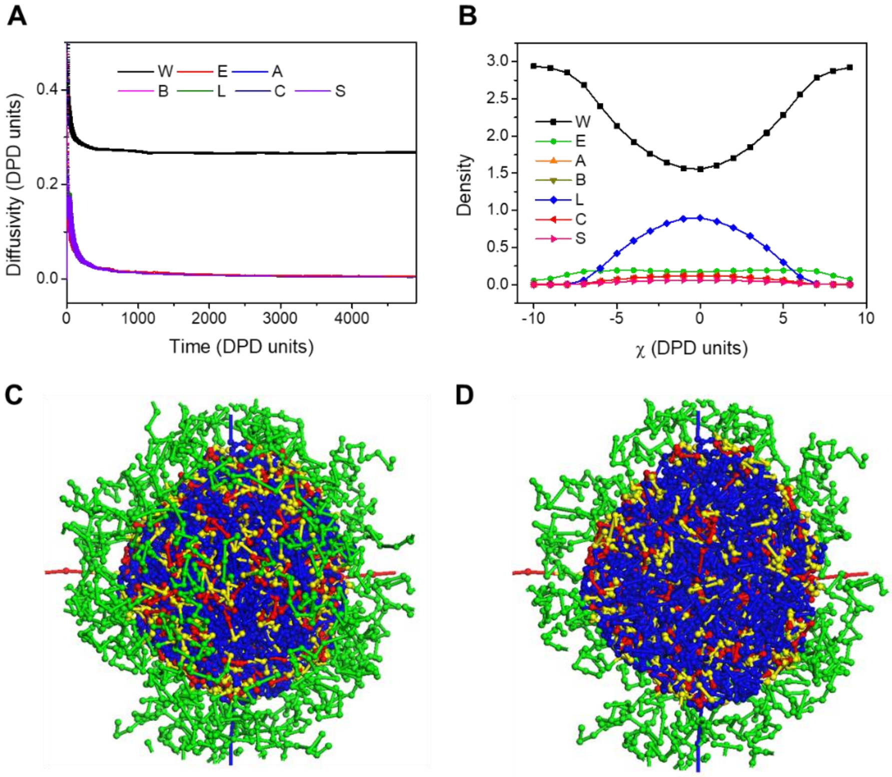

Figure S7. Computational simulation of MPU micelles. (A) Evolution of the diffusion coefficient of assembled MPU systems against the simulation steps. (B) Density profiles of MPU micelles. (C) Front view and (D) cross-sectional view of MPU micelles from DPD simulations. Color code: blue, PCL; green, PEG; red, Cys-PA; yellow, LDI.

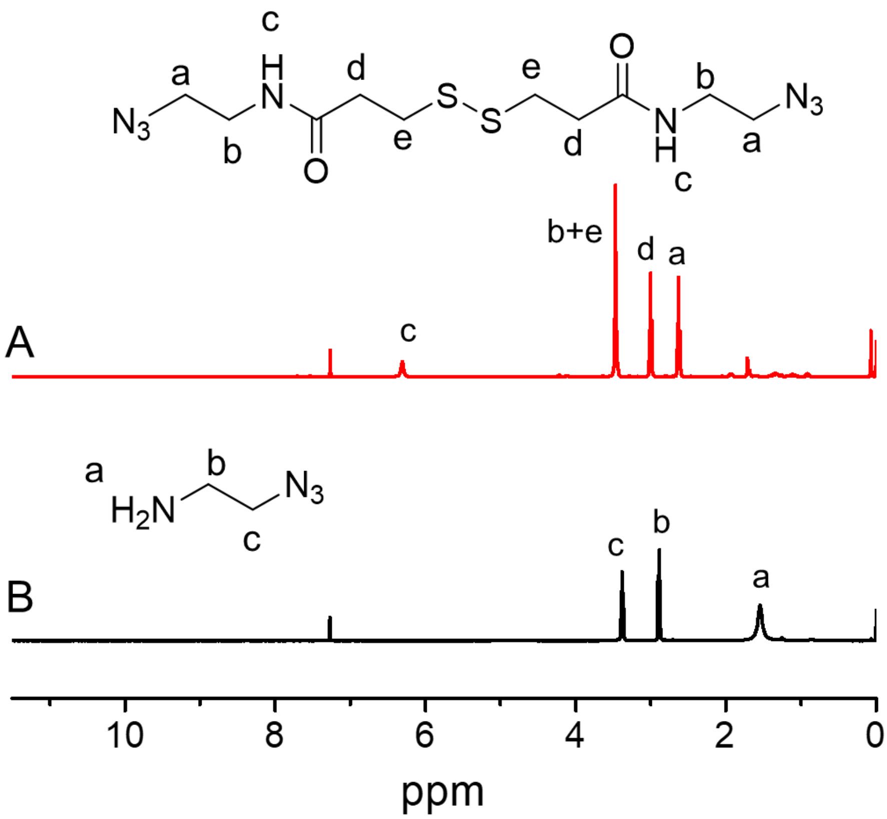

Figure S8. 400 MHz ^{1}H NMR spectra of SS-N_3 (A) and Az-EA (B) in $CDCl_3$.

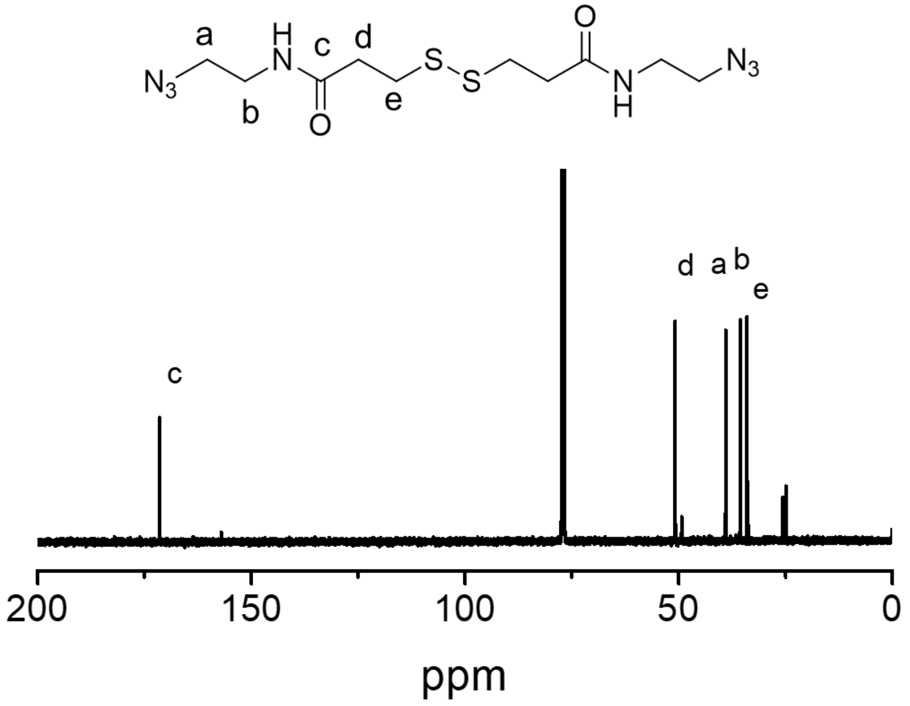

Figure S9. 400 MHz ^{13}C NMR spectrum of N_3EA-TDPA-N_3EA in $CDCl_3$.

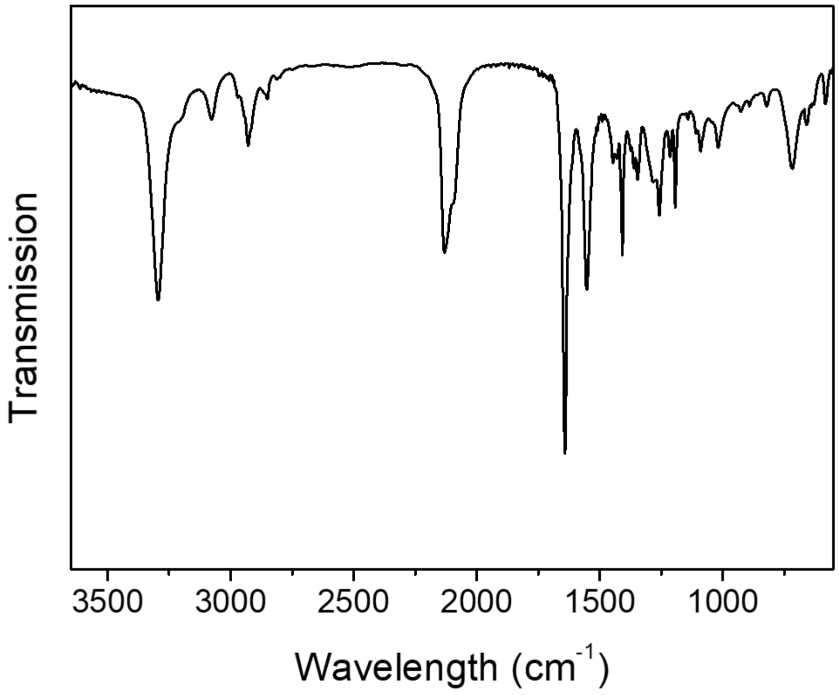

Figure S10. FTIR spectrum of SS-N_3.

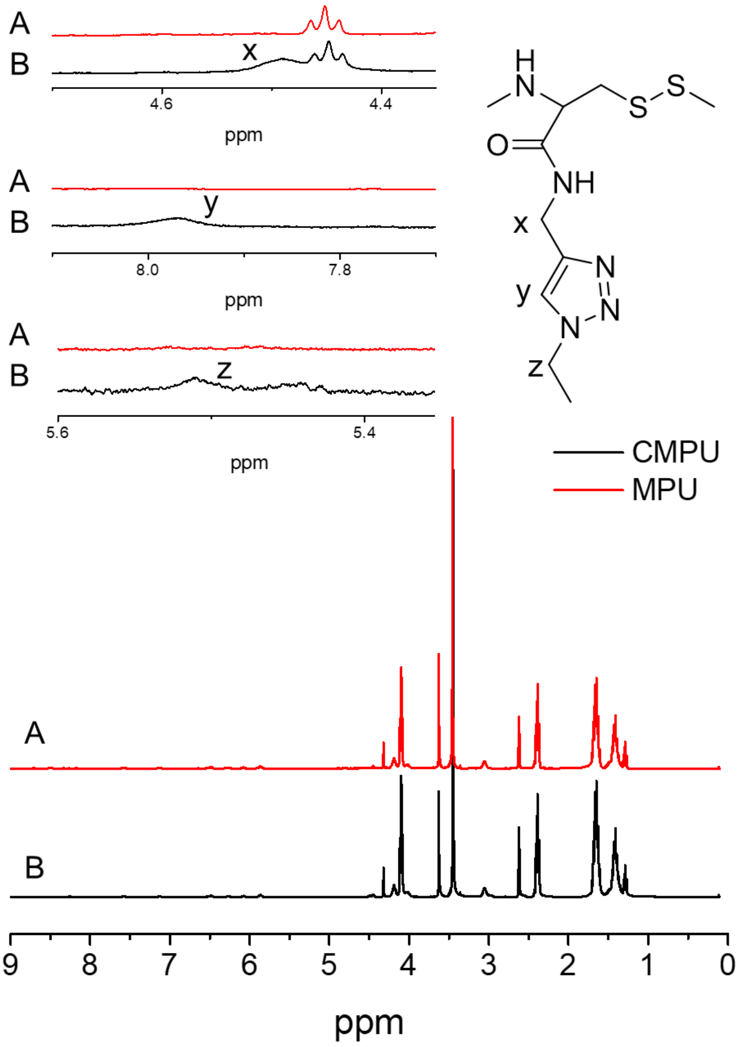

Figure S11. 400 MHz ^{1}H NMR spectra of multiblock polyurethane before (MPU, A) and after (CMPU, B) crosslinking recorded in DMSO-*d*6.

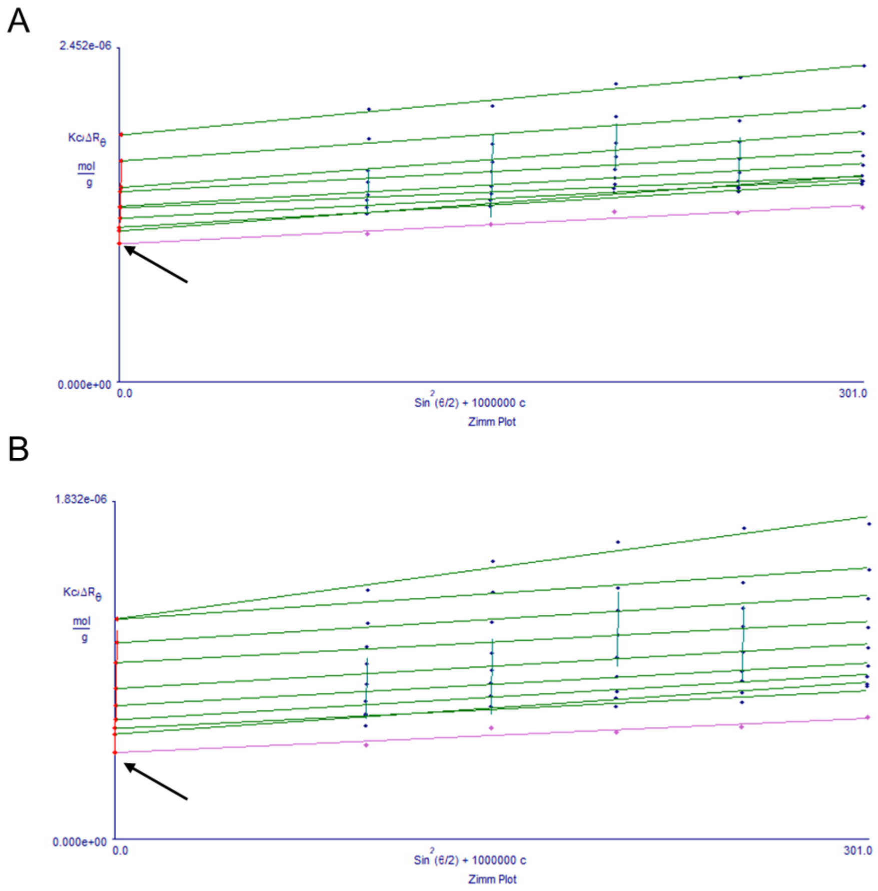

Figure S12. Zimm plots of MPU micelles before (A) and after (B) cross-linking. The molecular weight of MPU micelles before and after cross-linking was 9.87×10^5 and 2.12×10^6 obtained from extrapolation to zero angle and zero concentration (intercept) shown by the arrows.

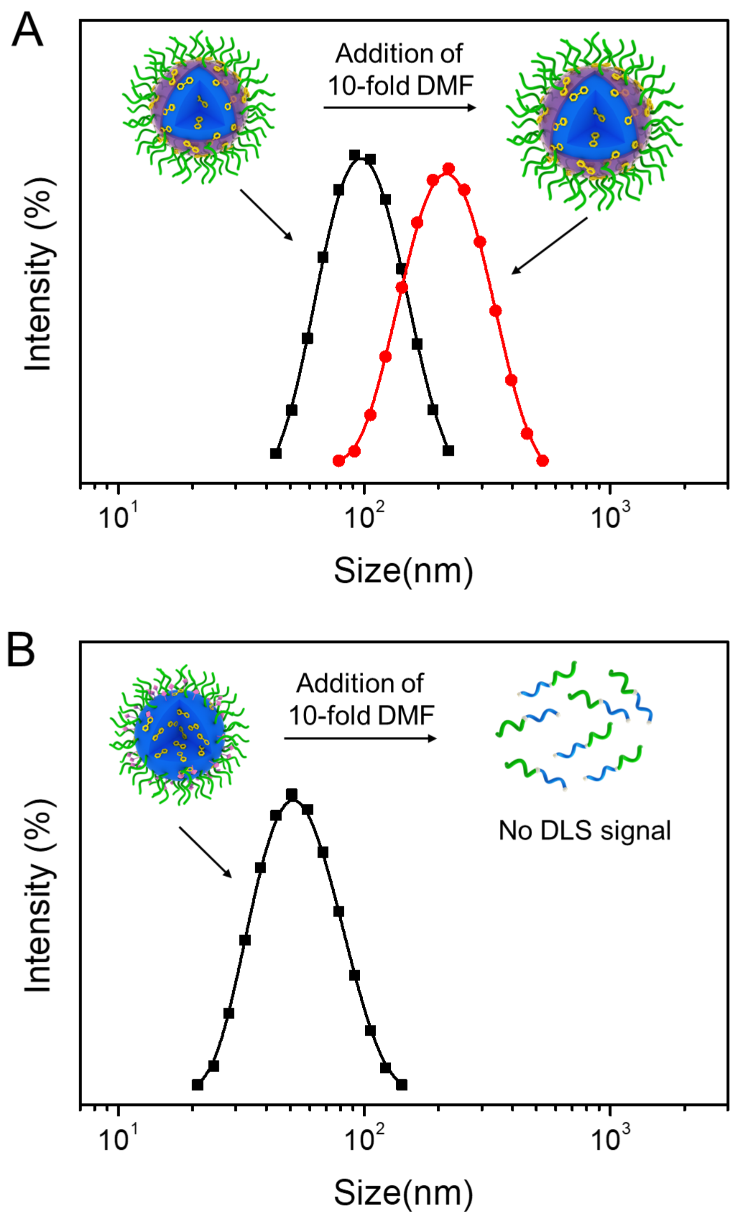

Figure S13. Distributions of hydrodynamic diameters of (A) CMPU and (b) MPU micelles in water before and after 10-fold dilution with DMF. The size of CMPU micelles increased from 103.7 nm to 197.0 nm in the presence of DMF. The DLS signal couldn't be detected in the presence of DMF.

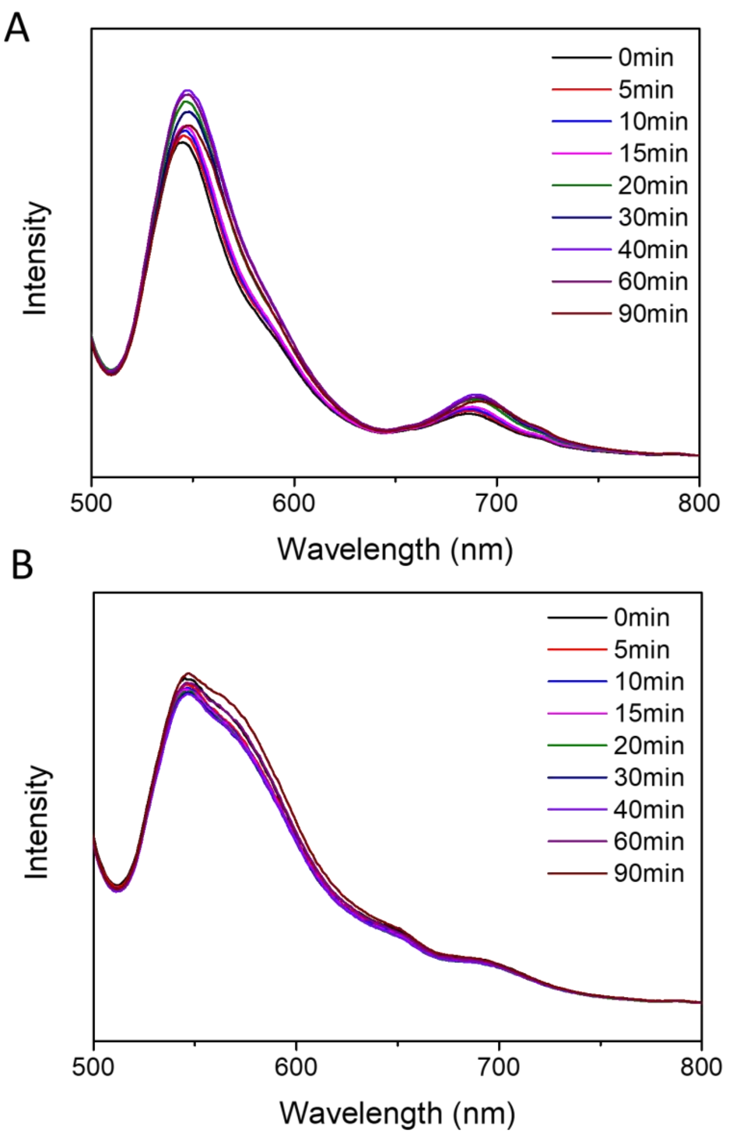

Figure S14. Fluorescence emission spectra (λ_{ex} = 480 nm) of mixture of DOX@MPU and Cy5@MPU (A), and mixture of DOX@CMPU and Cy5@CMPU (B) at different times.

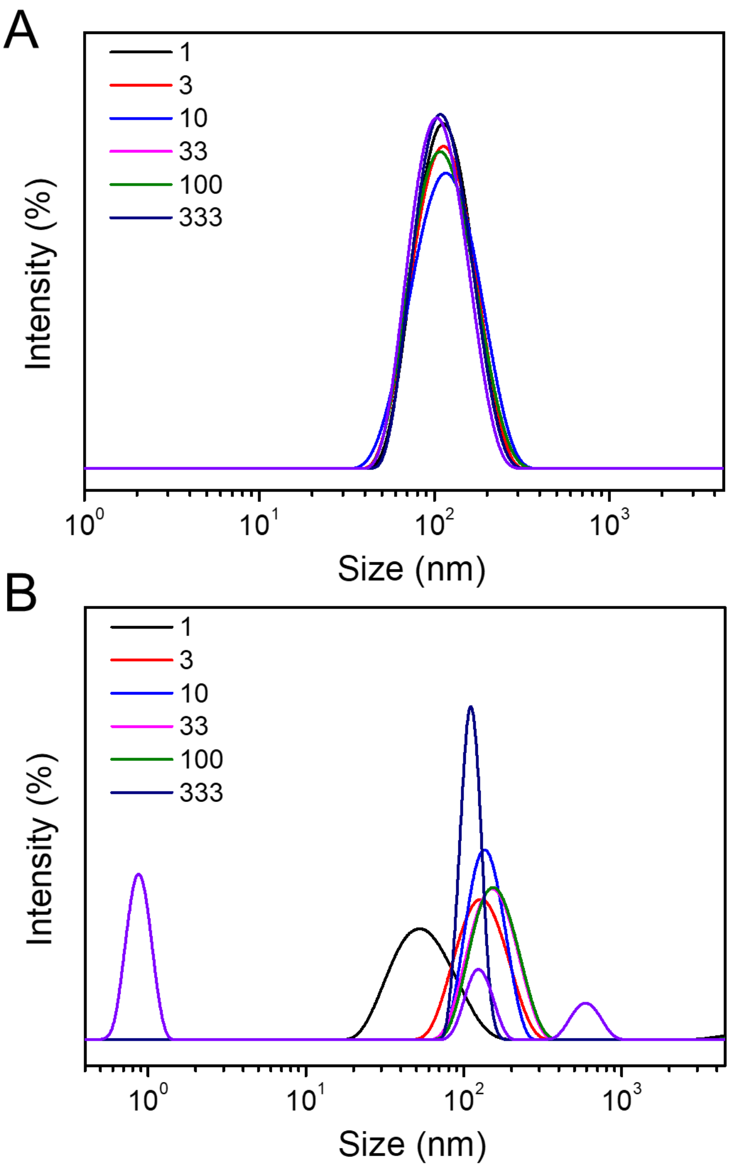

Figure S15. Size distributions of CMPU micelles (A) and MPU micelles (B) diluted with PBS for different times. The arabic numbers in the figure indicate dilution factors.

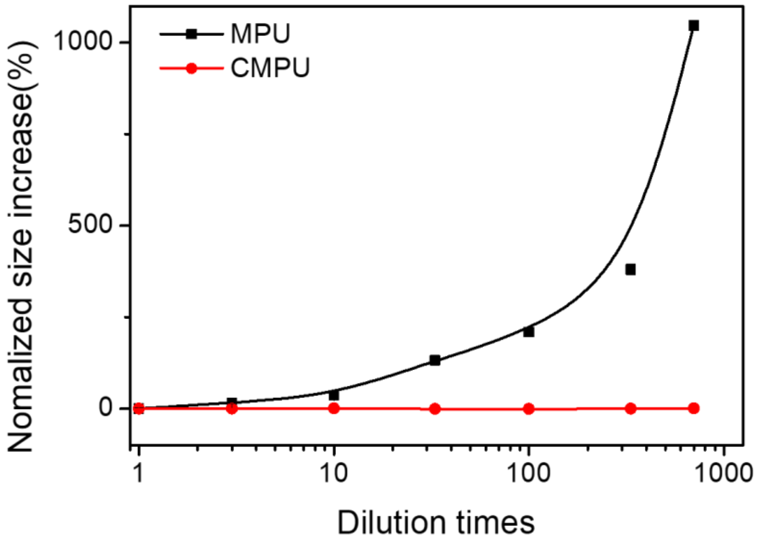

Figure S16. Normalized increase in size of MPU micelles before and after crosslinking upon dilution with PBS for different times.

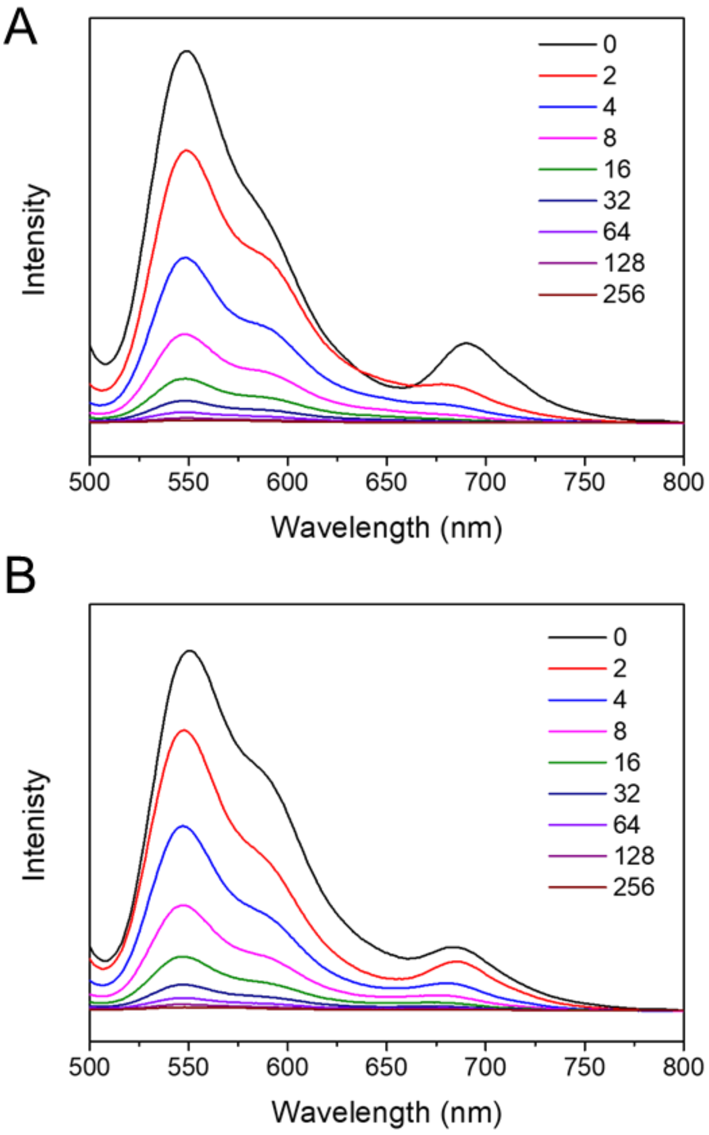

Figure S17. Fluorescence emission spectra (λ_{ex} = 480.0 nm) of DOX+Cy5@MPU (A) and DOX+Cy5@CMPU micelles (B) under different diluted times. The arabic numbers in the figure indicate dilution factors.

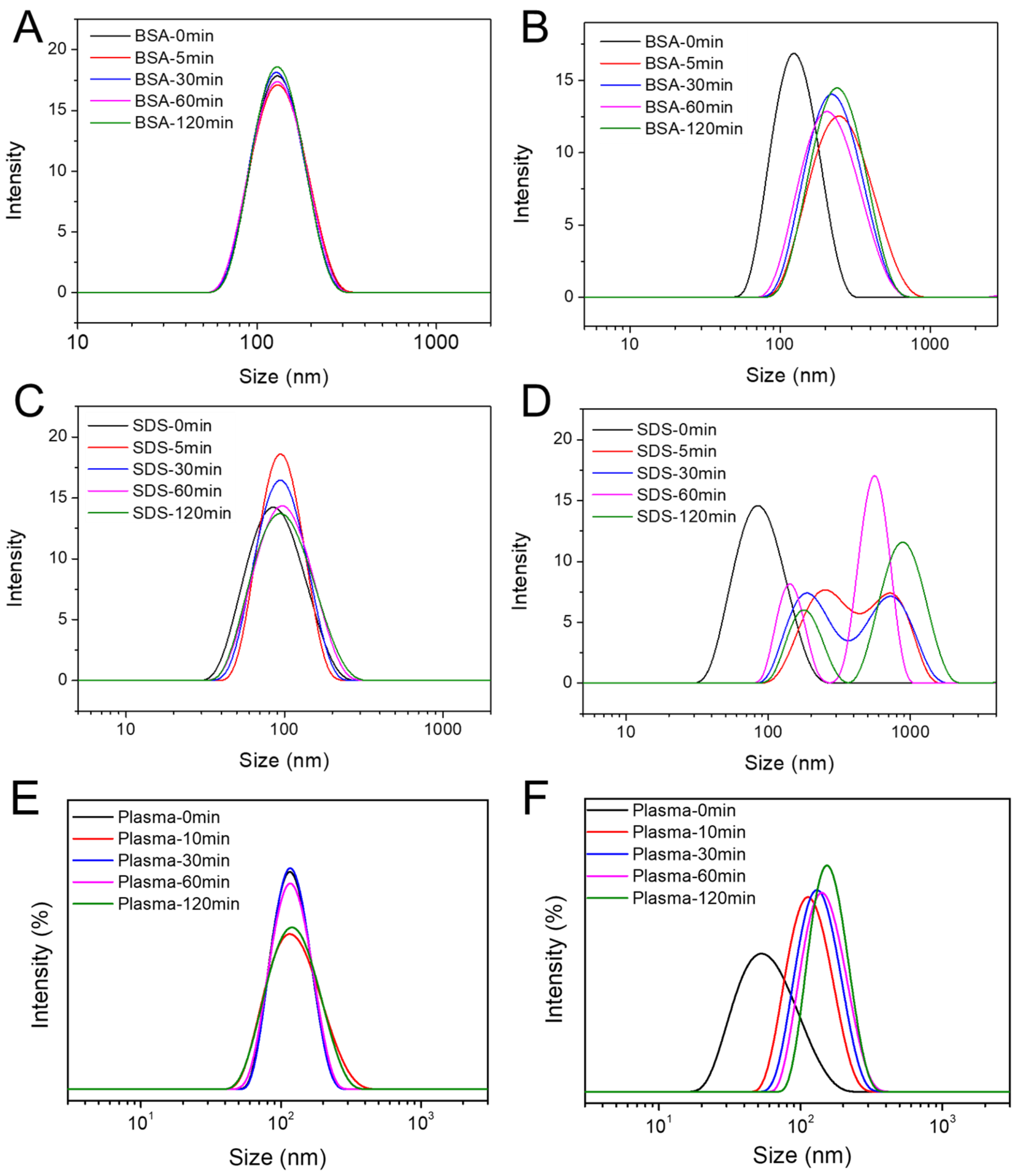

Figure S18. Size distributions of CMPU (A, C, E) and MPU (B, D, F) micelles in the presence of BSA (A, B), SDS (C, D) and fetal bovine serum (E, F) at different times.

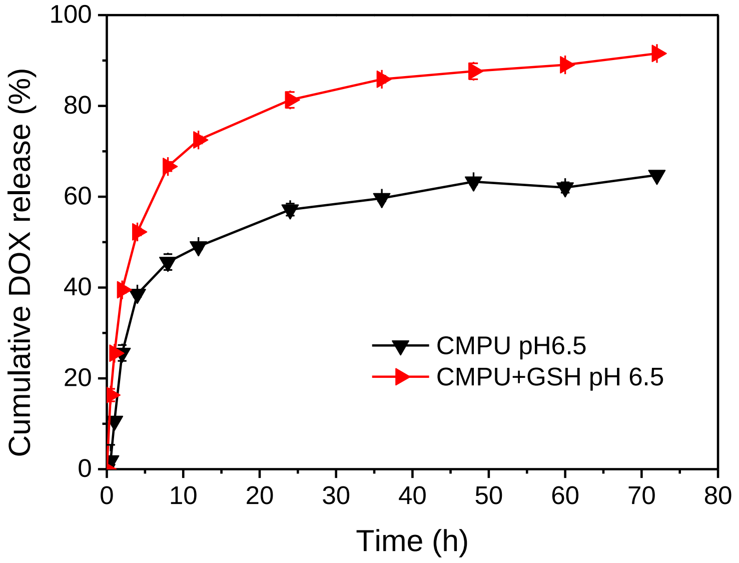

Figure S19. Cumulative release of DOX from CMPU micelles in PBS solutions (10 mM, pH 6.5) with or without GSH (10 mM).

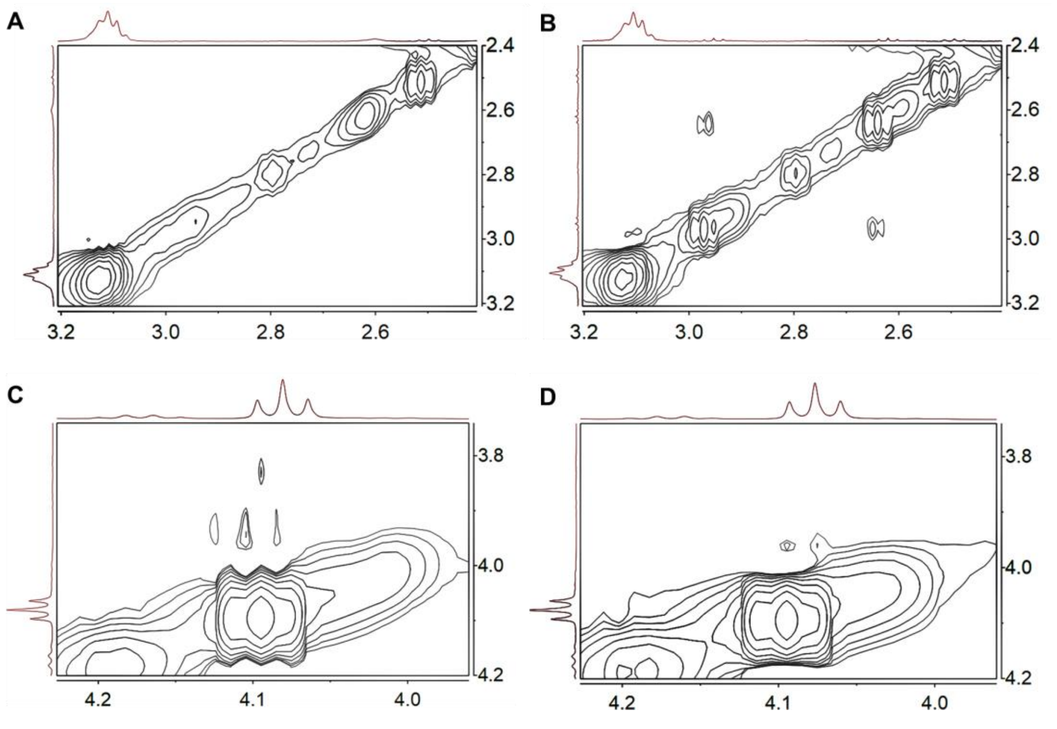

Figure S20. ^{1}H–^{1}H NOESY spectra of MPU micelles in CD_4O before (A, C) and after (B, D) crosslinking.

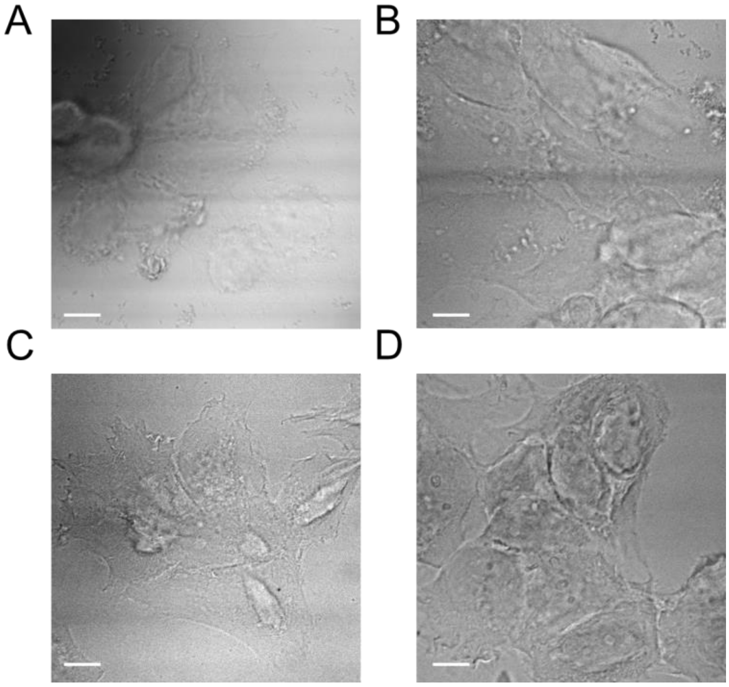

Figure S21. Bright field CLSM images of MCF-7 cells after cultures with DOX+Cy5@CMPU (A, B) and DOX+Cy5@MPU (C, D) for 1 h (A, C) and 4 h (B, D), respectively. The scale bars are 10 μm. The fluorescent images are shown in Figure 3A.

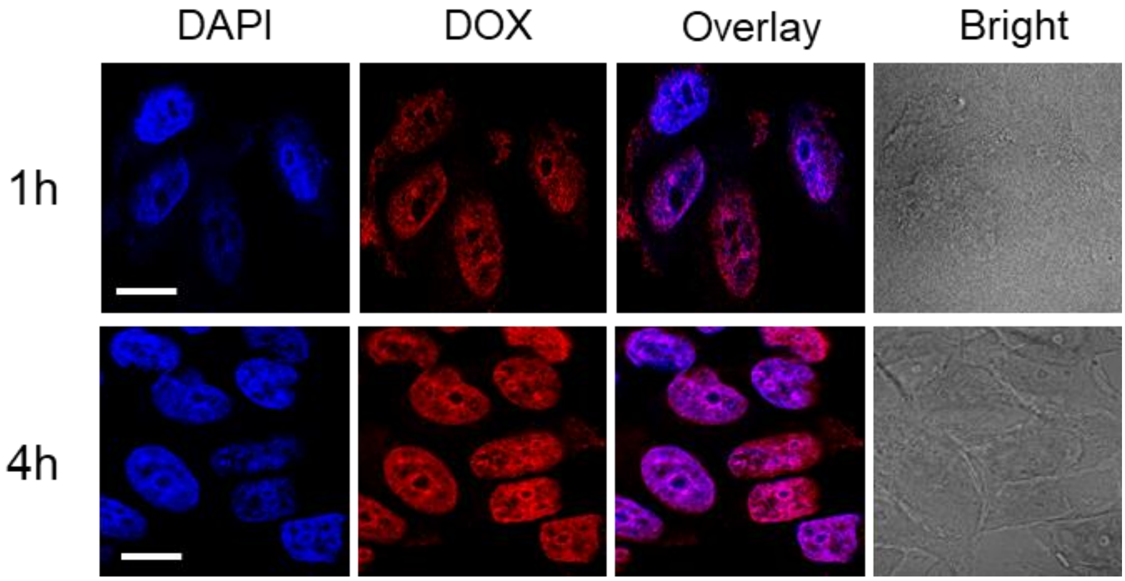

Figure S22. CLSM images of MCF-7 cells cultured with free DOX for 1 h and 4 h. The scale bars are 10 µm.

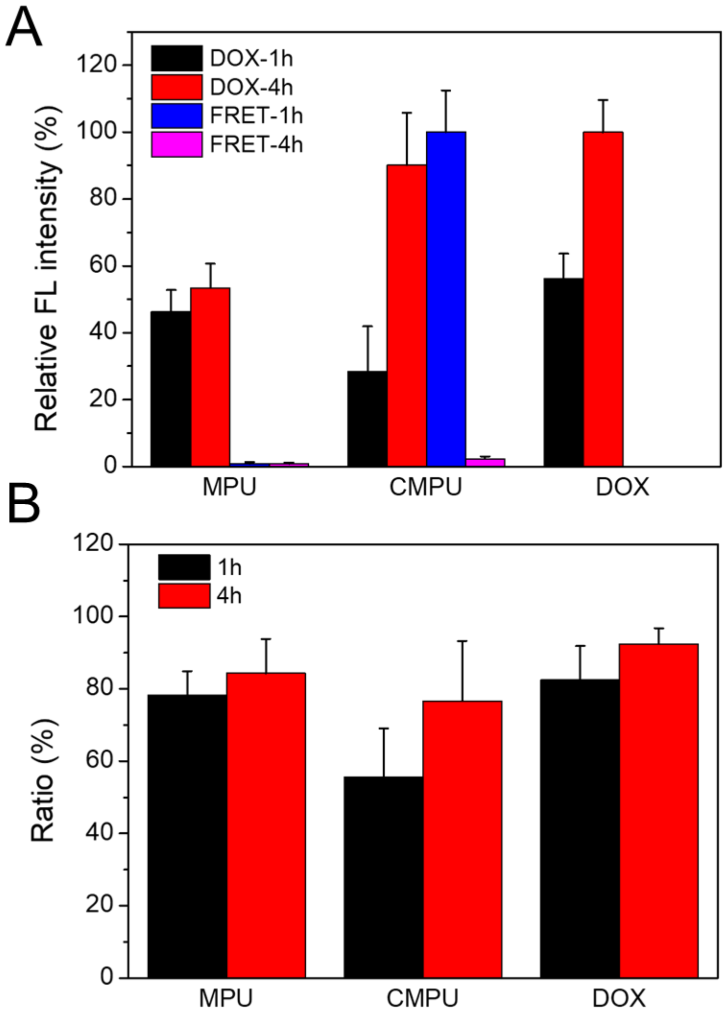

Figure S23. (A) The mean fluorescence intensity of DOX and FRET channels in MCF-7 tumor cells treated with different formulations for different times. (B) Proportion of DOX fluorescence intensity in nucleus of MCF-7 cells treated with different formulations for different times.

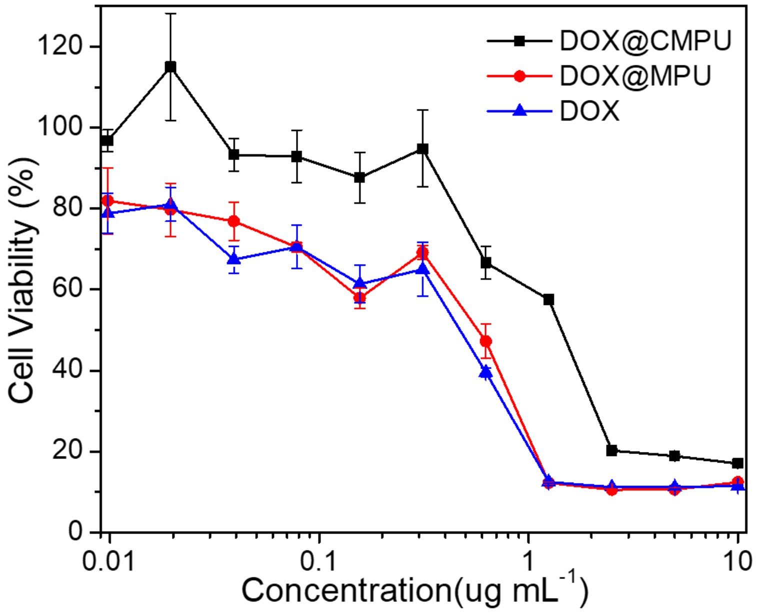

Figure S24. Cell viability of drug-sensitive MCF-7 cells incubated with DOX@CMPU an DOX@MPU micelles for 24 h with different concentrations of DOX, setting free DOX as a control.

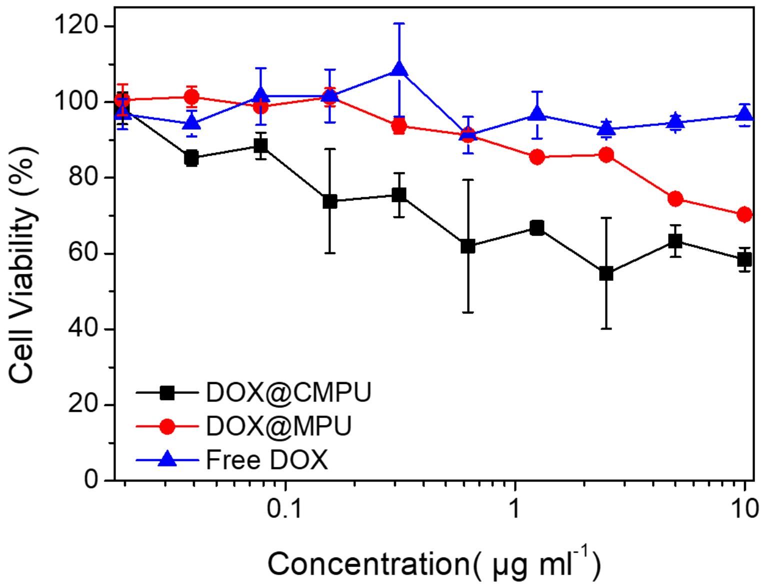

Figure S25. Cell viability of drug-resistant MCF-7 cells incubated with DOX@CMPU or DOX@MPU for 24 h with different concentrations of DOX, setting free DOX as a control.

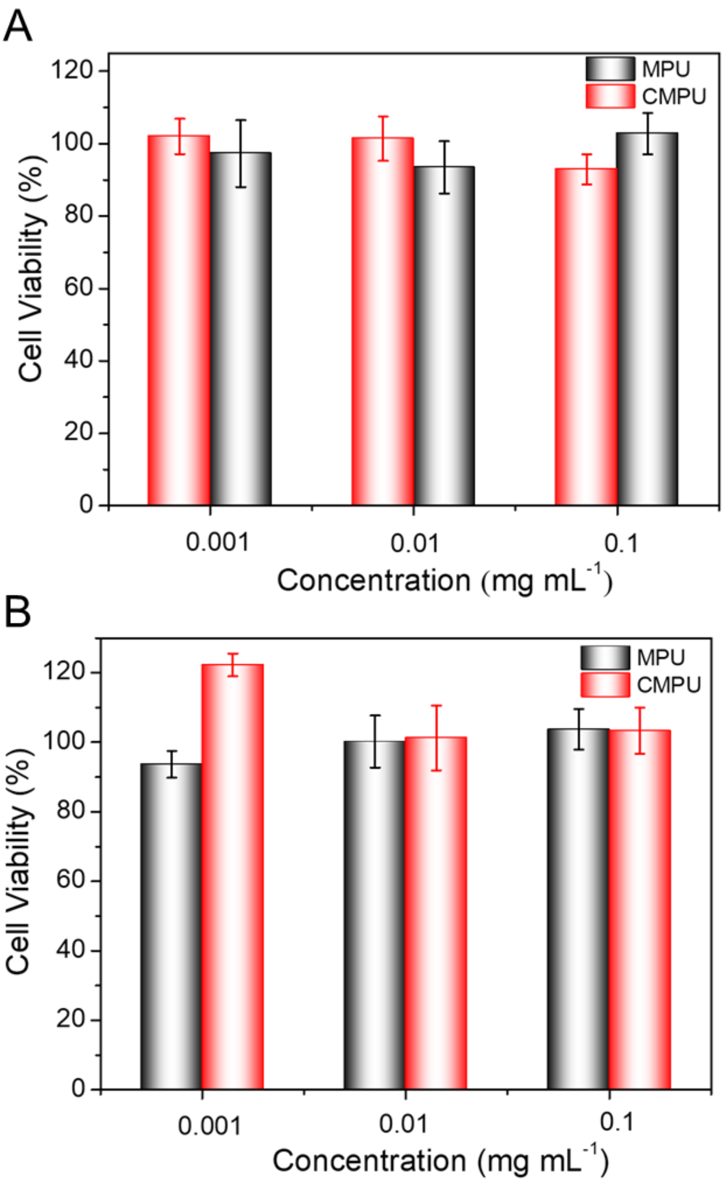

Figure S26. Cell viability of L929 mouse fibroblasts after incubation with drug-free MPU and CMPU micelles at different concentrations for 24 h (A) and 72 h (B).

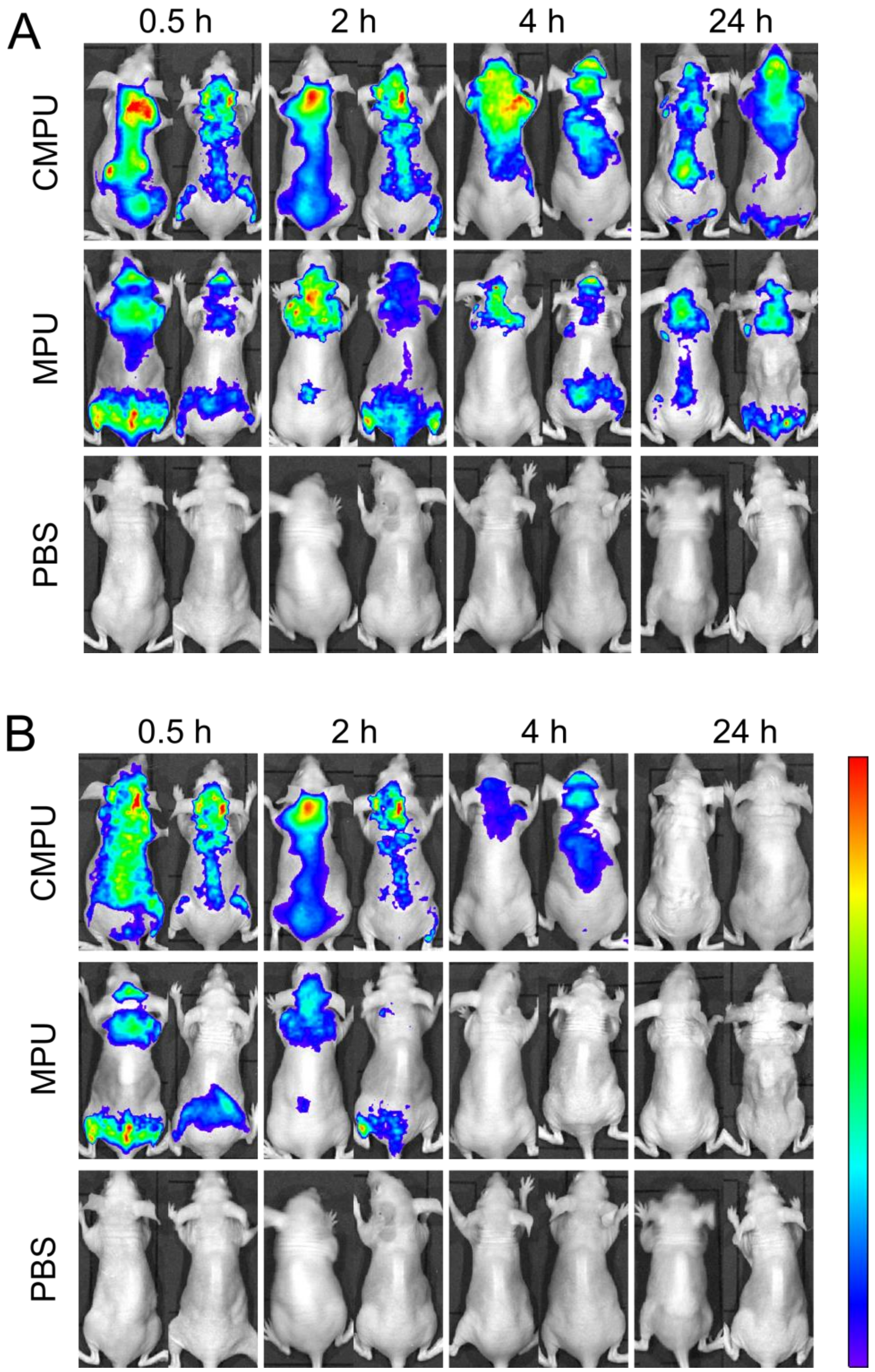

Figure S27. In vivo imaging of MCF-7 tumor-bearing mice at different times after intravenous injection of DOX+Cy5@MPU and DOX+Cy5@CMPU micelles. Mice receiving saline were set as control. (A) donor fluorescence channel, λ_{ex} = 480 nm, λ_{em} = 600 nm. (B) FRET fluorescence channel, λ_{ex} = 480 nm, λ_{em} =700 nm.

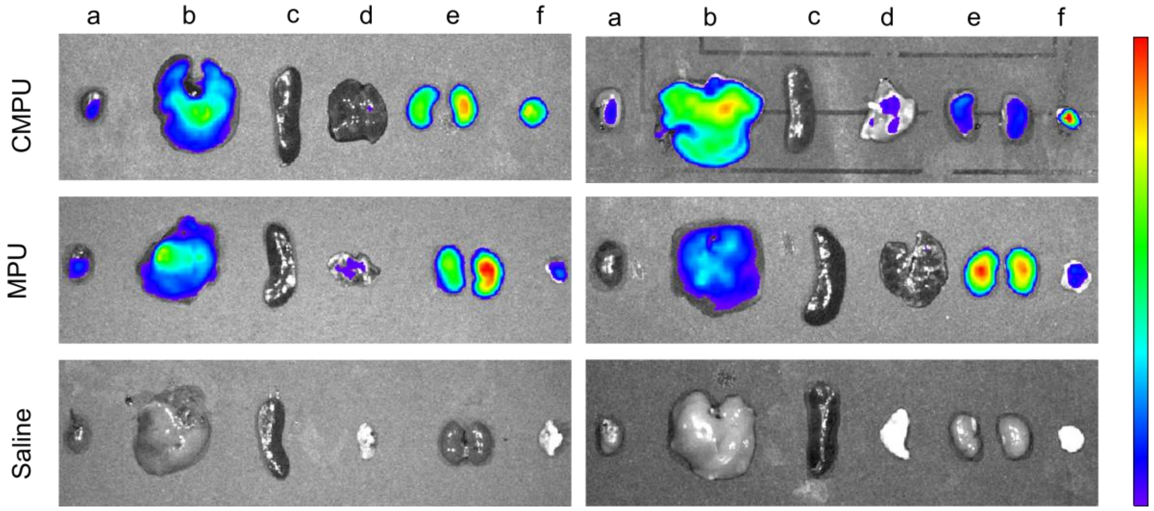

Figure S28. The *ex vivo* imaging of major organs and tumors of nude mice bearing MCF-7 tumors at 24 h post-injection of DOX+Cy5@MPU and DOX+Cy5@CMPU micelles (λ_{ex} = 480 nm, λ_{em} = 600 nm), where a, b, c, d, e and f represent heart, liver, spleen, lung, kidney and tumor, respectively.

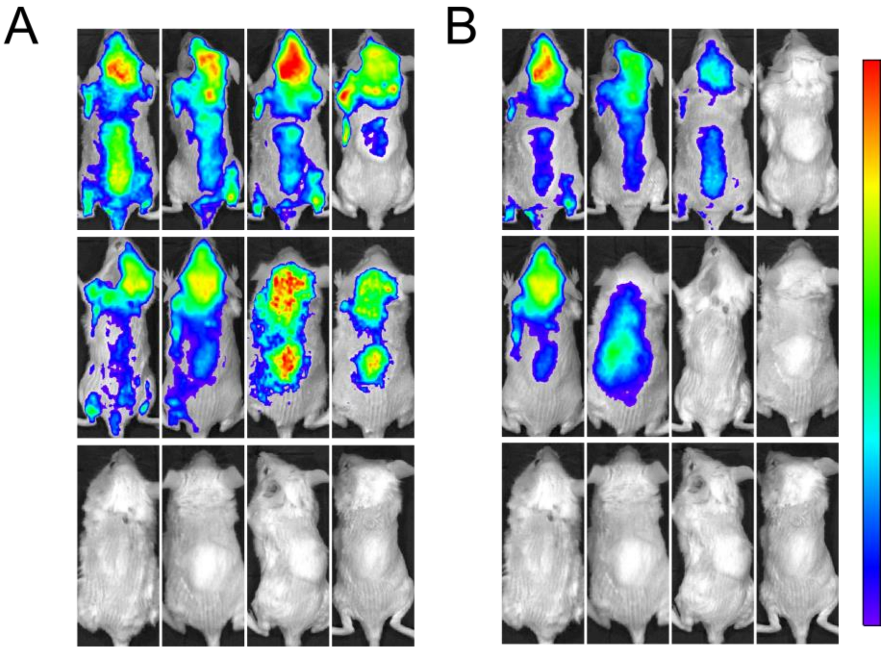

Figure S29. *In vivo* imaging of 4T1 tumor-bearing KM mice at different times after intravenous injection of DOX+Cy5@MPU and DOX+Cy5@CMPU micelles. Mice receiving saline were set as control. (A) donor fluorescence channel, λ_{ex} = 480 nm, λ_{em} =600 nm. (B) FRET fluorescence channel, λ_{ex} = 480 nm, λ_{em} =700 nm.

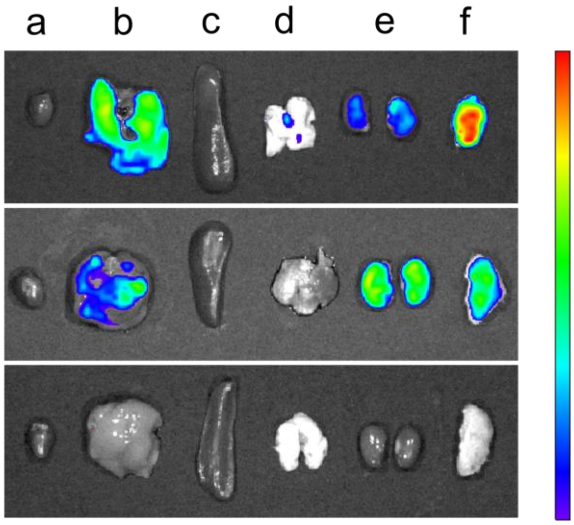

Figure S30. The *ex vivo* imaging of major organs and tumors of KM mice bearing 4T1 tumors at 24 h post-injection of DOX+Cy5@MPU and DOX+Cy5@CMPU micelles (λ_{ex} = 480 nm, λ_{em} = 600 nm), where a, b, c, d, e and f represent heart, liver, spleen, lung, kidney and tumor, respectively.

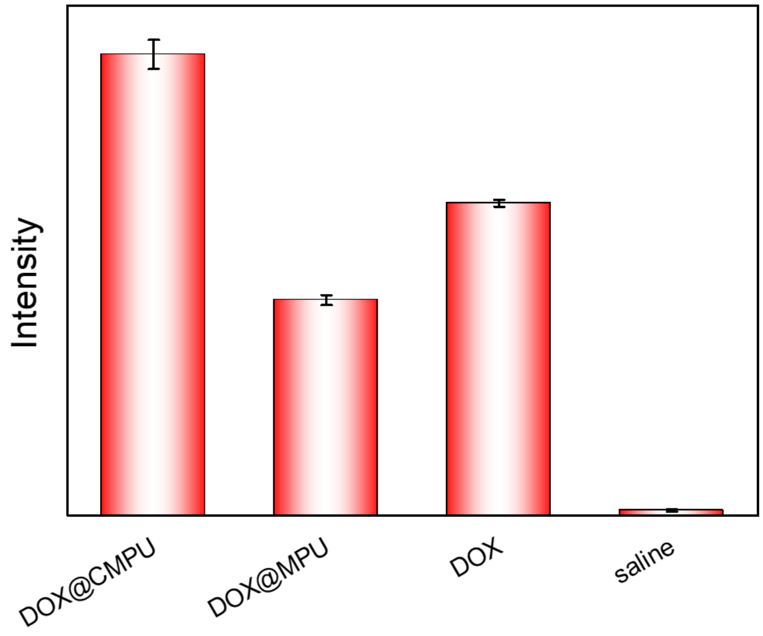

Figure S31. Immunofluorescence intensity of TUNEL immunostained tumor sections. The intensity was calculated with an ImageJ software.

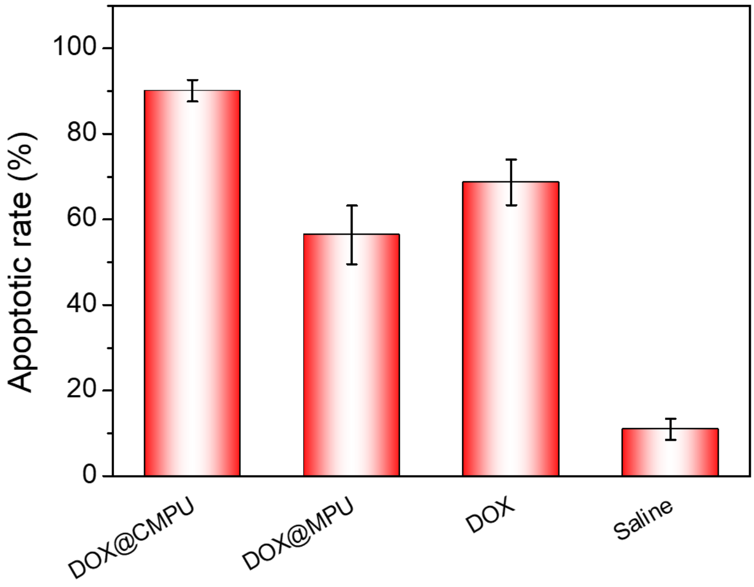

Figure S32. The percentage of apoptotic cells obtained from Ki67 immunostained tumor sections. The apoptotic cells were counted with an Image-Pro Plus software.

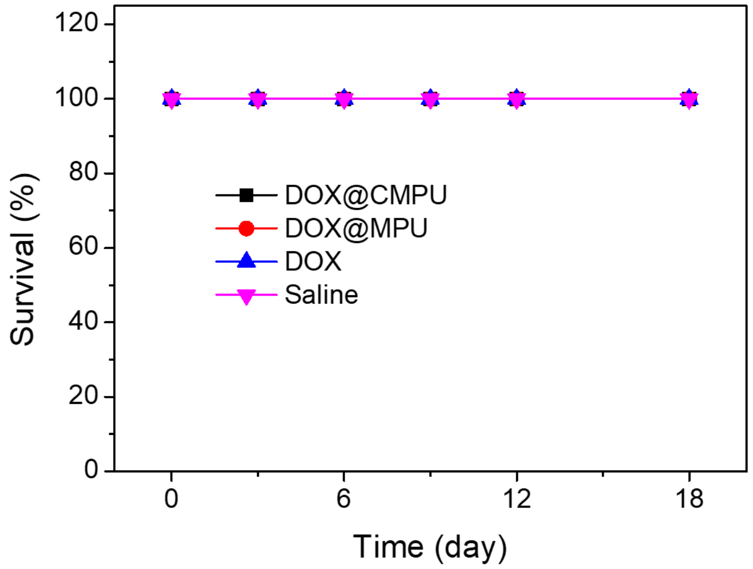

Figure S33. Survival rate of mice after injection of different formulations.

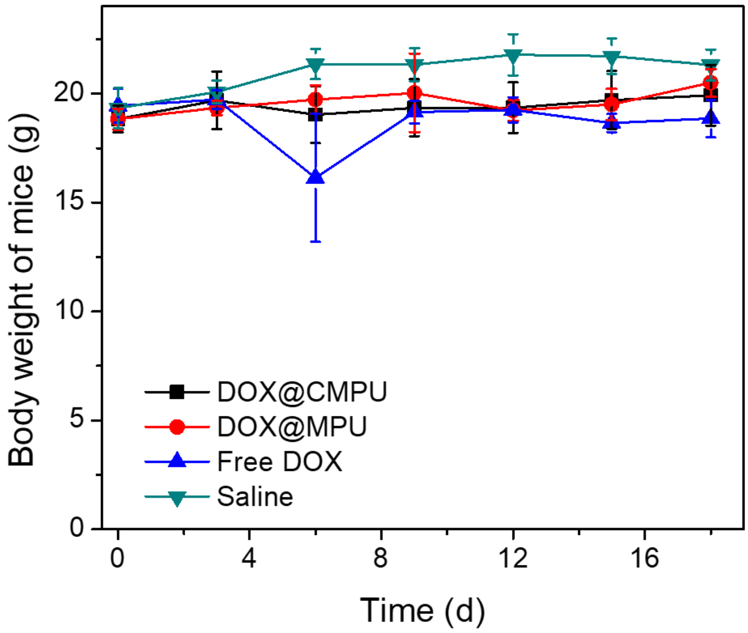

Figure S34. Body weight of mice after injection of different formulations.

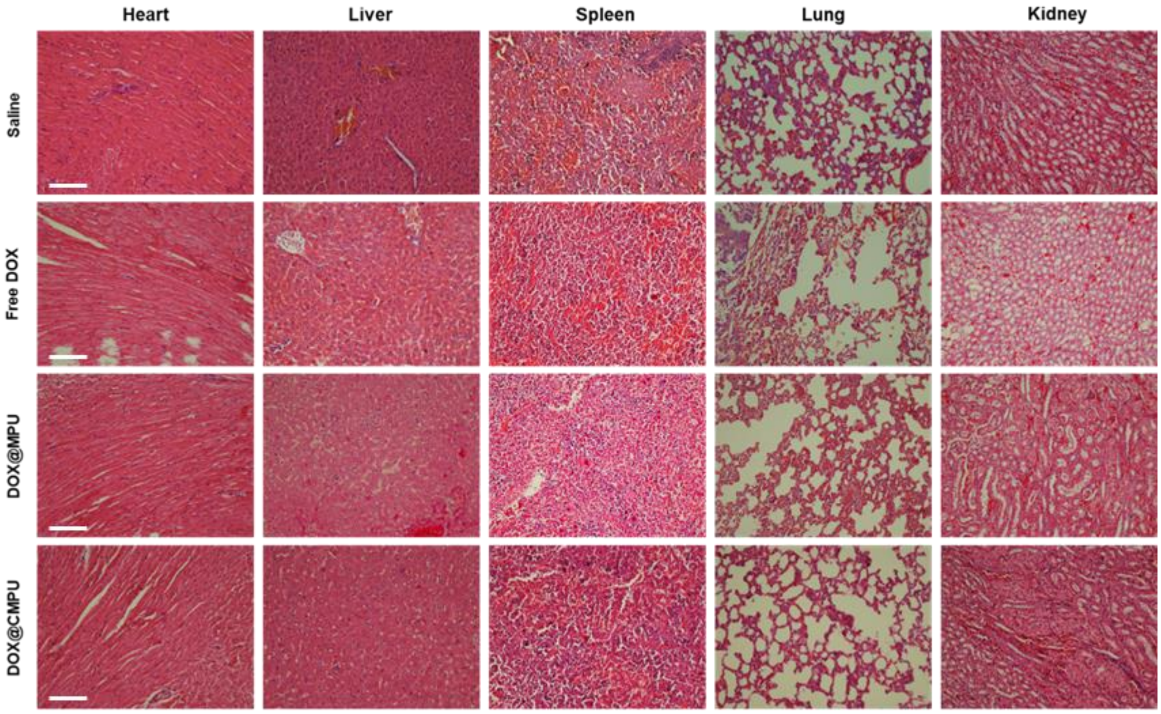

Figure S35. H&E staining analysis of the major organs (heart, liver, spleen, lung, kidney) of the mice after different treatments. The scale bars are 100 μm.

Table S1. Characterization of MPU assemblies before and after crosslink.

Sample[a]	Size (nm) [b]	Zeta potential (mv)[b]	N_{agg}[c]
MPU	52.8	-9.3	13.91
0.5CMPU	68.8	-14.7	---
10CMPU	111.0	-16.0	30.62

[a] Samples are denoted according to the feed ratios of SS-Az, where 0.5 and 10 indicate the equivalence of azide groups against alkyne sites.

[b] Size and zeta potential (ZP) determined using a Zetasizer Nano ZS instrument (Malvern Instruments Ltd., UK) at an angle of 90°.

[c] Aggregation numbers (N_{agg}) obtained from SLS measurement (Brookhaven Instruments BI-200SM goniometer).

Table S2. Interaction parameters a_{ij} in MPU micelle system.

	W	E	A	B	L	C	S
W	25.00						
E	25.99	25.00					
A	120.47	103.81	25.00				
B	114.07	100.06	25.08	25.00			
L	135.62	118.30	25.63	25.21	25.00		
C	114.96	99.94	25.01	25.04	25.48	25.00	
S	145.52	130.17	28.27	27.03	26.10	27.79	25.00

Supporting references

[1] N. Song, M. Ding, Z. Pan, J. Li, L. Zhou, H. Tan, Q. Fu, *Biomacromolecules* **2013**, *14*, 4407.

[2] J. Wei, X. Shuai, R. Wang, X. He, Y. Li, M. Ding, J. Li, H. Tan, Q. Fu, *Biomaterials* **2017**, *145*, 138.

[3] P. J. Hoogerbrugge, J. M. V. A. Koelman, *Europhys. Lett.* **1992**, *19*, 155.

[4] J. M. V. A. Koelman, P. J. Hoogerbrugge, *Europhys. Lett.* **1993**, *21*, 363.

[5] P. Español, P. Warren, *Europhys. Lett.* **1995**, *30*, 191.

[6] X. D. Guo, J. P. K. Tan, S. H. Kim, L. J. Zhang, Y. Zhang, J. L. Hedrick, Y. Y. Yang, Y. Qian, *Biomaterials* **2009**, *30*, 6556.

[7] M. Ding, X. He, Z. Wang, J. Li, H. Tan, H. Deng, Q. Fu, Q. Gu, *Biomaterials* **2011**, *32*, 9515.

[8] Z. Wang, J. Li, H. Tan, X. Zhang, Q. Fu, *Mol. Simul.* **2009**, *35*, 638.

[9] J. Li, Y. Chen, Z. Wang, M. Ding, H. Tan, Q. Fu, X. Jiang, *Langmuir* **2011**, *27*, 10859.

[10] M. Ding, N. Song, X. He, J. Li, L. Zhou, H. Tan, Q. Fu, Q. Gu, *ACS Nano* **2013**, *7*, 1918.

[11] Z. Pan, L. Yu, N. Song, L. Zhou, J. Li, M. Ding, H. Tan, Q. Fu, *Polym. Chem.* **2014**, *5*, 2901.
